# The Gestational Diabetes Antenatal Milk Expression (GAME) trial: effects on breastfeeding outcomes and maternal mental health—A randomised controlled trial

**DOI:** 10.1016/j.eclinm.2026.104130

**Published:** 2026-08-05

**Authors:** Yu Ngai Leung, Pui Hing Chau, Ying Dai, Christine Chi Oi Lam, Wing Cheong Leung, Kris Yuet Wan Lok

**Affiliations:** a School of Nursing, The University of Hong Kong, Hong Kong, China; b Department of Obstetrics & Gynaecology, Kwong Wah Hospital, Hong Kong; c Department of Obstetrics & Gynaecology, Queen Elizabeth Hospital, Hong Kong

**Keywords:** Gestational diabetes mellitus, Breastfeeding, Antenatal milk expression, Randomised controlled trial

## Abstract

**Background:**

Women with Gestational Diabetes Mellitus (GDM) are at increased risk of delayed lactogenesis and suboptimal breastfeeding. Although antenatal milk expression (AME) may facilitate lactation, its safety and efficacy in this population are not established. We aimed to evaluate the effects of a structured AME programme on breastfeeding and maternal psychosocial outcomes in women with GDM.

**Methods:**

We conducted a two-centre, parallel-group, assessor-blinded, randomised controlled trial at two public antenatal clinics in Hong Kong from May 10, 2023, to May 10, 2024. Two hundred and fourty-six pregnant women with singleton pregnancy and diagnosis of GDM at 34–36 weeks’ gestation were randomly assigned (1:1) to receive the Gestational Diabetes and Antenatal Milk Expression (GAME) intervention, comprising education and support, or standard care. The primary outcome was exclusive breastfeeding across the postpartum period assessed at three timepoints: hospital discharge, four weeks postpartum, and eight weeks postpartum. Secondary outcomes included maternal depressive symptoms, breastfeeding self-efficacy, and breastfeeding self-regulation. Analysis was by intention-to-treat using Generalised Linear Mixed Models. This trial is registered with ClinicalTrials.gov, NCT05851651.

**Findings:**

Over the 8-week postpartum period, the proportion of women exclusively breastfeeding in the intervention group was higher than in control group at discharge (16.3% vs. 8.9%; aOR 2.28 [95% CI 1.01–5.14]), four weeks (30.9% vs. 20.3%; aOR 2.00 [1.07–3.74]), and eight weeks (29.3% vs. 25.2%; aOR 1.37 [0.73–2.56]). The intervention group demonstrated significantly higher odds of exclusive breastfeeding over time (adjusted odds ratio 2.43 [95% CI 1.17–5.04], p < 0.05). Regarding secondary outcomes, the intervention group reported significantly lower maternal depressive symptoms at 8 weeks postpartum (mean difference −1.74 [95% CI −2.94 to −0.54], p = 0.004) and higher breastfeeding self-efficacy at 8 weeks postpartum (mean difference 3.30 [95% CI 0.12–6.48], p = 0.042). Rates of any breastfeeding did not differ significantly between groups at any time point (aOR 0.68 [95% CI 0.21–2.20]). Neonatal hypoglycaemia and gestational age at birth did not differ between groups.

**Interpretation:**

The GAME programme significantly increased exclusive breastfeeding, improved breastfeeding self-efficacy, and reduced maternal depressive symptoms in women with GDM, with no added perinatal risk. Integrating structured AME into routine antenatal care could improve both infant feeding and maternal psychological well-being in this high-risk population.

**Funding:**

Kowloon Central Cluster Research Grant (KCC/RG/G/2223-A02), Hospital Authority and Seed Funding for Basic Research (2202100786) at The University of Hong Kong.

**Research in context**

**Evidence before this study**

AME, the practice of hand-expressing colostrum in late pregnancy, has been investigated primarily in general obstetric populations or women with pre-existing diabetes. We searched MEDLINE, Embase, and Cochrane Central databases of controlled trials for articles published up to November 2025 using the terms including “antenatal milk expression,” “colostrum collection,” “gestational diabetes mellitus,” and “breastfeeding outcomes.” Studies from high-income countries have suggested that AME may improve lactation readiness and exclusive breastfeeding rates, and potentially reduce neonatal hypoglycaemia. However, the existing evidence is limited by small, heterogeneous studies with diverse intervention protocols, and a paucity of data from public healthcare settings in Asia. Crucially, no randomised controlled trials had evaluated a structured AME programme specifically for women with GDM within routine antenatal care, nor had any study prioritised its effects on maternal mental health.

**Added value of this study**

To our knowledge, this is the first assessor blinded, randomised controlled trial to evaluate a culturally adapted, structured AME programme for women with GDM in a real-world public antenatal setting. The GAME intervention significantly increased the rate of exclusive breastfeeding rates at 4 weeks postpartum by over 14 percentage points and substantially reduced maternal depressive symptoms at 8 weeks postpartum, compared with standard care. The programme was safe, with no increased risk of neonatal hypoglycaemia or earlier birth, and demonstrated high feasibility and acceptability among participants. This study provides robust evidence that a low resource intervention, integrated into routine care, can simultaneously improve key infant feeding and maternal psychological outcomes in a high-risk population.

**Implications of all the available evidence**

Cumulatively, evidence suggests that structured AME is feasible, safe and potentially effective for improving breastfeeding outcomes. For a population at heightened risk of breastfeeding difficulties and postpartum depression, this approach offers a practical strategy to empower mothers and promote early lactation success. Health systems should consider incorporating protocols for supported AME education into standard antenatal care pathways for women with GDM. Future research should investigate the long-term maternal and child metabolic outcomes, cost-effectiveness, and optimal implementation strategies across diverse cultural and healthcare contexts.

## Introduction

GDM is a major and growing global public health concern. According to the International Diabetes Federation, hyperglycemia in pregnancy affects approximately one in six live births (15.6%) worldwide, the majority of which is attributable to GDM^1^; GDM alone is estimated to complicate 14% of pregnancies globally, with prevalence reaching 20–30% in high-risk populations.^1^ Beyond its immediate perinatal complications, GDM confers a substantial long-term health burden, increasing a woman's risk of developing type 2 diabetes and cardiovascular disease seven to ten-fold^2^ and predisposing her offspring to childhood obesity and metabolic disorders.^3^

Breastfeeding offers a critical opportunity to mitigate these intergenerational risks. Lactation improves maternal glucose metabolism and insulin sensitivity, reducing the progression to type 2 diabetes in both mother and child.^4^, ^5^, ^6^ Yet, women with GDM face significant barriers to breastfeeding success. They are at high risk of delayed lactogenesis II, while their infants are vulnerable to neonatal hypoglycaemia, often leading to formula supplementation and separation for treatment.^7^ Psychologically, these women frequently experience lower breastfeeding self-efficacy and higher rates of postpartum depressive symptoms.^8^ Consequently, despite its proven benefits, exclusive breastfeeding rates in this population remain low.

AME, the hand expression and storage of colostrum from around 36 weeks of pregnancy, has emerged as a potential intervention to overcome these barriers. By providing a ready supply of colostrum, AME could facilitate the management of neonatal hypoglycaemia without resorting to formula, while potentially enhancing maternal confidence and physiological preparedness for lactation.^9^ However, clinical guidance on AME remains inconsistent and is not supported by robust evidence. Concerns about the risk of preterm labour persist, and data on its effects, particularly on maternal mental health are scarce.^10^ Furthermore, in many Asian contexts, including Hong Kong, cultural reservations and the absence of standardised protocols limits its practice.

To address these evidence gaps, we conducted the GAME trial. This assessor-blinded, randomised controlled trial aimed to evaluate the efficacy and safety of a structured AME programme for Chinese women with GDM in Hong Kong. We hypothesised that the intervention would improve rates of exclusive breastfeeding and maternal psychosocial outcomes without increasing perinatal risks.

## Methods

### Study design and participants

Eligible participants were pregnant women aged 18 years or older, with a singleton pregnancy, diagnosed with GDM between 34 and 36 weeks’ gestation according to the International Association of Diabetes and Pregnancy Study Groups (IADPSG) criteria (adopted by the Hong Kong College of Obstetricians and Gynaecologists).^11^ Exclusion criteria included pre-existing diabetes, multiple gestation, contraindications to breastfeeding, polyhydramnios, history of preterm labour, or other obstetric conditions classified as high-risk. All participants provided written informed consent prior to enrolment.

**Fig. 1:**
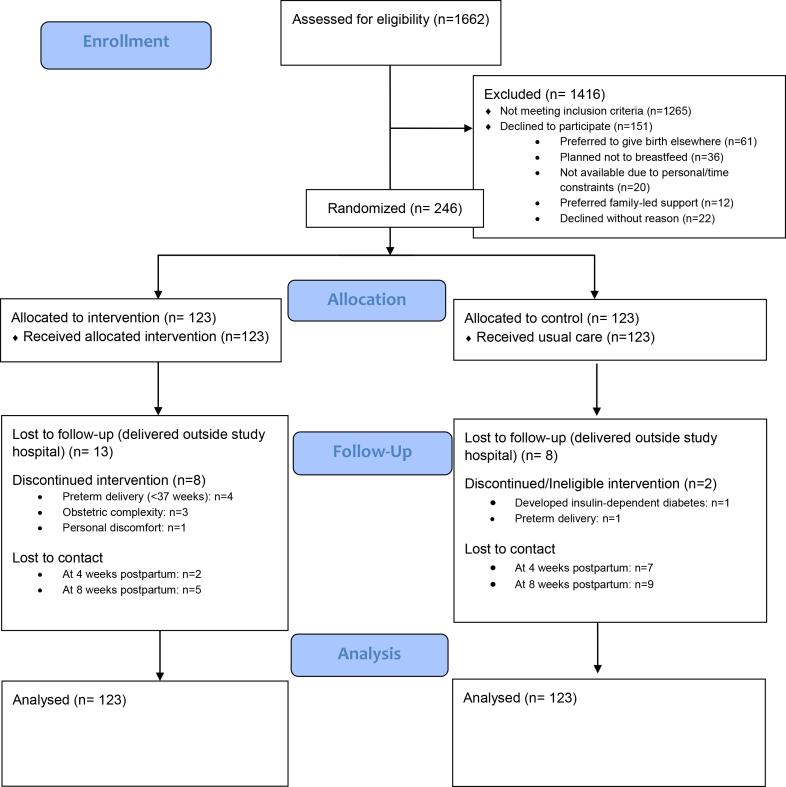
**CONSORT flow diagram**.

We conducted a two-centre, parallel-group, assessor-blinded, randomised controlled trial at two public antenatal clinics in Hong Kong from 10th May, 2023 to 10th May, 2024. A total of 1662 women were assessed for eligibility. Of these, 1416 were excluded, primarily because they did not meet the inclusion criteria (n = 1265) or declined to participate (n = 151). The remaining 246 participants were randomised (1:1) to either the intervention group (n = 123) or the control group (n = 123). In accordance with the intention-to-treat principle, all 246 participants (123 in the intervention group and 123 in the control group) were included in the final analysis. Participant flow through the trial is shown in the CONSORT diagram (Fig. 1).

### Ethics

The study was approved by the Institutional Review Boards of the University of Hong Kong/Hong Kong Western Cluster (UW21-364) and the Hospital Authority Kowloon Centre Cluster/Kowloon East Research Ethics Committee (KC/KE-21-0119/ER-1). The trial was registered with ClinicalTrials.gov, NCT05851651 on 17th April 2023.

### Randomisation and masking

Participants were randomly assigned (1:1) to the intervention (GAME programme plus standard care) or control group (standard care only). Randomisation was performed using a computer-generated sequence with permuted block sizes, stratified by study site. Allocation concealment was ensured using sequentially numbered, sealed opaque envelopes prepared by an independent administrator not involved in recruitment. Outcome assessors and data analysts were masked to group assignment. Given the nature of the behavioural intervention, participants and the clinicians delivering it could not be masked.

### Procedures

Participants in the intervention group received the GAME programme, a structured, theory-based intervention designed to support antenatal hand expression and improve breastfeeding self-efficacy. The programme was based adapted from the protocol used in the Diabetes and Antenatal Milk Expressing (DAME) randomised controlled trial and international guidelines.^10^^,^^12^ The intervention comprised a 45-min face-to-face education session at 36 weeks’ gestation delivered by a trained midwife or International Board-Certified Lactation Consultant (IBCLC), followed by weekly telephone support until delivery. The education session followed a standardised curriculum and script, which included: (i) discussion of the benefits of breastfeeding and AME for women with GDM, including reduced risk of neonatal hypoglycaemia and long-term maternal Type 2 diabetes risk; (ii) review of common GDM-related breastfeeding challenges and strategies to overcome them; (iii) goal-setting and barrier-identification exercises, in which participants identified personal strengths, anticipated difficulties, and coping strategies; (iv) viewing of a standardised video demonstrating hand expression of colostrum, breastfeeding positioning, and attachment; (v) hands-on coaching in hand expression under the guidance of the midwife or IBCLC, with instruction on positioning, attachment, and hygiene protocols; and (vi) provision of a GDM-specific breastfeeding leaflet and a GAME factsheet, which included a QR code linked to the hand-expression and positioning videos for home reference.

The session utilised a GAME factsheet, a GDM-specific breastfeeding leaflet, and a video demonstrating hand expression techniques. Participants practised hand expression under the guidance of the midwife and received instruction on positioning, attachment, and hygiene protocols. Participants were instructed to begin antenatal colostrum expression from 37 weeks' gestation, aiming for up to 10 min total per session twice daily, and to bring any expressed colostrum to the labour ward.

Telephone follow-up calls (weekly from 37 to 39 weeks' gestation) used a semi-structured checklist to: reinforce key messages from the education session, troubleshoot difficulties with hand expression or positioning, assess adherence, and provide motivational support. All intervention facilitators underwent standardised training using a GAME facilitator manual and were observed periodically to ensure consistent delivery; a session checklist was completed for each participant. The control group received standard antenatal care plus the same GDM-specific breastfeeding leaflet but did not receive the structured education session, video demonstration, hands-on practice, or telephone support.

The GDM-specific breastfeeding leaflet was developed by a certified lactation consultant and midwife, and covered: (i) the importance of early colostrum feeding to prevent neonatal hypoglycaemia in infants of diabetic mothers; (ii) recommended techniques and timing of early breastfeeding or hand expression in women with GDM; (iii) maternal blood glucose monitoring during breastfeeding; and (iv) the reduced risk of future Type 2 diabetes mellitus associated with lactation. These points were also reinforced verbally during the education session and follow-up telephone calls.

Women were encouraged to begin antenatal colostrum expression from 37 weeks' gestation, aiming for twice-daily sessions of up to 10 min total per session (combined for both breasts). A colostrum collection kit was provided, and participants were instructed to freeze expressed colostrum and bring it to the hospital upon admission for delivery. To reinforce adherence, research midwives and lactation consultants conducted two follow-up telephone calls at 38 weeks and 39–40 weeks’ gestation (approximately 20 min each) to assess progress, address barriers, and provide feedback. Participants in the control group received routine antenatal care without specific instruction on antenatal milk expression. Patients and the public were not involved in the design, conduct, or reporting of this trial.

### Outcomes

The primary outcomes were any breastfeeding and exclusive breastfeeding, assessed at baseline (on hospital discharge), four weeks postpartum, and eight weeks postpartum. Exclusive breastfeeding was defined according to WHO/UNICEF criteria as the infant receiving only breast milk (direct breastfeeding and/or expressed maternal breast milk, including expressed antenatal or postnatal colostrum/milk), with no other liquids or solids except prescribed medications, oral rehydration solution, or vitamin/mineral supplements.^13^ Donor human milk and infant formula were not considered exclusive breastfeeding. Infants who received any formula, water, glucose water, or complementary foods prior to the assessment time point were classified as not exclusively breastfed. Feeding practices were assessed prospectively using a structured feeding diary completed by mothers and verified by trained research assistants during telephone follow-up interviews conducted at each time point.

Secondary outcomes included breastfeeding self-efficacy, breastfeeding self-regulation, and maternal depressive symptoms, all of which were assessed at baseline, four weeks postpartum, and eight weeks postpartum, respectively. Breastfeeding self-efficacy was measured using the Chinese version of Breastfeeding Self-Efficacy Scale–Short Form (BSES-SF), a 14-item instrument designed to assess a mother's confidence in her ability to breastfeed.^14^ Items are rated on a 5-point Likert scale ranging from 1 (“not at all confident”) to 5 (“always confident”), yielding a total score between 14 and 70, with higher scores indicating greater breastfeeding self-efficacy. The scale has demonstrated excellent internal consistency (Cronbach α = 0.95)^14^; in the current study, Cronbach α coefficients were 0.95, 0.96, and 0.96 at baseline, four weeks, and eight weeks, respectively.

Breastfeeding self-regulation was assessed using the Breastfeeding Self-Regulation Questionnaire (BSRQ). Grounded in self-determination theory, this 22-item questionnaire evaluates the extent to which breastfeeding behaviour is driven by autonomous motivation (i.e., engaging in breastfeeding because it is personally valued and enjoyable) versus controlled motivation (i.e., breastfeeding due to external pressure, guilt or obligation). Higher scores on the autonomous regulation subscale indicate greater internal motivation, which has been consistently associated with higher rates of breastfeeding initiation, exclusivity, and longer breastfeeding duration in previous research.^15^^,^^16^ Conversely, higher controlled regulation scores reflect motivation driven by external factors and have been linked to earlier breastfeeding cessation. The original Cronbach α for the BSRQ is 0.86^16^; in this study, the internal consistency was high, with Cronbach α values of 0.898, 0.89, and 0.914 across the three time points.

Maternal depressive symptoms were evaluated using the Chinese version of Edinburgh Postnatal Depression Scale (EPDS).^17^ This 10-item screening tool asks participants to rate the frequency of symptoms over the past seven days on a 4-point scale (0–3), resulting in a total score of 0–30, where higher scores indicate a greater risk of depression. The internal consistency for this study was satisfactory, with Cronbach α coefficients of 0.76, 0.83, and 0.77 at baseline, four weeks, and eight weeks, respectively.

Neonatal outcomes included the incidence of hypoglycaemia (defined as blood glucose <2.6 mmol/L in the first 24 h), neonatal intensive care unit (NICU) admission, and length of stay. Maternal safety outcomes included the incidence of uterine contractions, preterm labour, or hospitalisation following the initiation of antenatal milk expression.

### Sample size calculation

The sample size was calculated *a priori* to detect a clinically meaningful difference in exclusive breastfeeding rates at the primary endpoint of 4 weeks postpartum, based on prior intervention studies.^18^ A systematic review has demonstrated that professional breastfeeding support can increase exclusive breastfeeding rates by up to 43% at 4–6 weeks postpartum.^19^ Conservatively assuming a baseline rate of 20% in the control group and an expected increase of 18% (to 38%) in the intervention group (Cohen's h = 0.40), a sample size of 123 participants per group provided greater than 80% power at a 5% significance level (two-sided). Our final sample of 246 participants satisfied this requirement. The primary outcome was analysed using Generalised Linear Mixed Models (GLMM), which accounted for repeated measurements across the three time points (discharge, 4 weeks, and 8 weeks postpartum) without requiring multiplicity adjustment for the primary endpoint, as the power calculation was based solely on the 4-week time point.

### Statistical analysis

Baseline characteristics and secondary outcomes were summarised as means and standard errors (SE) for continuous variables, and as frequencies and percentages for categorical variables. Between-group comparisons were performed using pooled t-tests for continuous data and pooled Chi-squared tests (or Fisher's exact tests for low cell counts) for categorical data.

Outcomes were analysed according to the intention-to-treat (ITT) principle. For the primary outcomes (any and exclusive breastfeeding rates), missing data were handled using a worst-case scenario approach, where missing values were assumed to indicate cessation of breastfeeding. To assess the robustness of this approach, we compared baseline characteristics between participants with complete and missing outcome data to evaluate the missing at random (MAR) assumption and conducted a complete-case sensitivity analysis excluding participants with any missing breastfeeding data. To examine the effects of the GAME intervention on primary breastfeeding outcomes across time, we used GLMM with a logit link function. Two pre-specified model structures were used for each outcome: (i) a main-effects model (group + time + covariates), which estimates the overall pooled treatment effect across the three time points and constitutes the primary analysis; (ii) a model additionally including the group-by-time interaction term, which constitutes the secondary/exploratory analysis and examines whether treatment effects varied across time points. Both model structures were pre-specified in the statistical analysis plan. The models included fixed effects for group, time, and the group-by-time interaction, with random intercepts for participants to account for within-subject correlation. Multivariable logistic regression was also performed to assess group differences at specific time points (baseline, four weeks, and eight weeks).

For secondary continuous outcomes (EPDS, BSES-SF, BSRQ), independent t-tests were used to assess mean differences at each time point, followed by Linear Mixed Models (LMM) with random intercepts to evaluate longitudinal trajectories over the full study period. All multivariable models (GLMM, Logistic Regression, and LMM) were adjusted for the following pre-specified covariates: maternal age, study site, maternal education, return to work postpartum, intention to breastfeed, parity, and breastfeeding support. Estimated marginal means and adjusted odds ratios (aOR) with 95% confidence intervals (CI) were calculated.

Normality of continuous outcomes was assessed using Q–Q plots and the Shapiro–Wilk test at each time point. EPDS scores showed significant right skew at all time points (Shapiro–Wilk p = 0.020 to p < 0.001). BSES-SF scores were approximately normally distributed (all p > 0.05). BSRQ scores were normal at baseline but showed mild departure from normality at later time points (p = 0.004–0.033). The LMM framework used for primary analysis is robust to moderate departures from normality, and with 123 participants per group, the Central Limit Theorem ensures reliable parametric inference. Q–Q plots and full Shapiro–Wilk results are reported in the Figure S1. Statistical significance was defined as a two-sided p-value less than 0.05. Analyses were performed using R (version 4.4.0) and SPSS version 26.0 (IBM Corp, Armonk, NY, USA).

### Role of funding source

The funder of the study had no role in study design, data collection, data analysis, data interpretation, or writing of the report.

## Results

### Participants and recruitment

#### Baseline characteristics

All 246 participants were Chinese women. Baseline sociodemographic and obstetric characteristics including race/ethnicity were well-balanced between the intervention and control groups (see Table 1). The study population had a mean maternal age of 34.6 years, and the majority of participants were multiparous (53.7%). Socioeconomic indicators, including education levels and employment status, were similarly distributed between the groups. Clinical characteristics regarding gestational diabetes were also comparable; on average, participants were diagnosed at 25.3 weeks gestation, and there were no significant differences in antenatal glycaemic control, including fasting and post-load glucose levels.

**Table 1:** Baseline demographic characteristics.

	Total	AME group	Control group
	(N = 246)	(n = 123)	(n = 123)
	Mean ± SD/n (%)	Mean ± SD/n (%)	Mean ± SD/n (%)
Age	34.6 (4.40)	34.6 (4.49)	34.6 (4.34)
Parity groups			
Multiparous	132 (53.7%)	64 (52.0%)	68 (55.3%)
Primiparous	114 (46.3%)	59 (48.0%)	55 (44.7%)
Marital status			
Married	232 (94.3%)	118 (95.9%)	114 (92.7%)
Unmarried	14 (5.7%)	5 (4.1%)	9 (7.3%)
Maternal education			
Diploma or sub-degree	48 (19.5%)	26 (21.1%)	22 (17.9%)
Primary or below	5 (2.0%)	2 (1.6%)	3 (2.4%)
Secondary	108 (43.9%)	54 (43.9%)	54 (43.9%)
Undergraduate or above	85 (34.6%)	41 (33.3%)	44 (35.8%)
Employment (Full Time)			
No	113 (45.9%)	54 (43.9%)	59 (48.0%)
Yes	133 (54.1%)	69 (56.1%)	64 (52.0%)
Household income per month (HKD)^a^			
<$20000	65 (26.4%)	35 (28.5%)	30 (24.4%)
$20000–$40000	107 (43.5%)	44 (35.8%)	63 (51.2%)
>$40000	74 (30.1%)	44 (35.8%)	30 (24.4%)
Intended breastfeeding duration			
<3 months	16 (6.5%)	7 (5.7%)	9 (7.3%)
3 to <6 months	114 (46.3%)	58 (47.2%)	56 (45.5%)
6 months to 1 year	85 (34.6%)	43 (35.0%)	42 (34.1%)
>1 year	31 (12.6%)	15 (12.2%)	16 (13.0%)
Breastfeeding support from family			
No opinion	23 (9.3%)	14 (11.4%)	9 (7.3%)
Not support	5 (2.0%)	2 (1.6%)	3 (2.4%)
Support	141 (57.3%)	71 (57.7%)	70 (56.9%)
Strongly support	77 (31.3%)	36 (29.3%)	41 (33.3%)
Return to work after baby born			
No	48 (19.5%)	21 (17.1%)	27 (22.0%)
Yes	136 (55.3%)	72 (58.5%)	64 (52.0%)
Not decided yet	62 (25.2%)	30 (24.4%)	32 (26.0%)
Smoking during pregnancy			
No	236 (95.9%)	118 (95.9%)	118 (95.9%)
Yes	10 (4.1%)	5 (4.1%)	5 (4.1%)
Previous gestational diabetes history			
No	91 (37.0%)	45 (36.6%)	46 (37.4%)
Yes	56 (22.8%)	27 (22.0%)	29 (23.6%)
Not applicable	99 (40.2%)	51 (41.5%)	48 (39.0%)
Previous children breastfeed			
No	24 (9.8%)	14 (11.4%)	10 (8.1%)
Yes	120 (48.8%)	57 (46.3%)	63 (51.2%)
Not applicable	102 (41.5%)	52 (42.3%)	50 (40.7%)
Past breastfeeding experience (duration)			
≤ 3 months	42 (17.1%)	22 (17.9%)	20 (16.3%)
3–6 months	22 (8.9%)	5 (4.1%)	17 (13.8%)
6 months to 1 year	26 (10.6%)	17 (13.8%)	9 (7.3%)
>1 year	34 (13.8%)	15 (12.2%)	19 (15.4%)
Not applicable	122 (49.6%)	64 (52.0%)	58 (47.2%)
Past infant feeding method			
Exclusive breastfeeding	38 (15.4%)	19 (15.4%)	19 (15.4%)
Breastfeeding with occasional supplement	44 (17.9%)	21 (17.1%)	23 (18.7%)
Half breastfeeding half formula	27 (11.0%)	11 (8.9%)	16 (13.0%)
Mostly infant formula	12 (4.9%)	8 (6.5%)	4 (3.3%)
Infant formula	12 (4.9%)	6 (4.9%)	6 (4.9%)
Not applicable	113 (45.9%)	58 (47.2%)	55 (44.7%)
Attended breastfeeding class(es)			
No	149 (60.6%)	76 (61.8%)	73 (59.3%)
Yes	97 (39.4%)	47 (38.2%)	50 (40.7%)
Clinical Characteristics			
Gestational age on recruitment (wk)	34.7 (2.87)	34.8 (1.87)	34.6 (3.60)
Body Height (cm)	159 (5.40)	159 (5.44)	158 (5.34)
BMI (kg/m^2^)	22.8 (3.26)	23.1 (3.48)	22.6 (3.00)
Random capillary blood glucose, mmol/L^b^	0.377 (1.51)	0.335 (1.54)	0.420 (1.50)
OGTT fasting glucose, mmol/L (antenatal)^b^	4.84 (0.552)	4.87 (0.563)	4.82 (0.541)
OGTT 2-h glucose, mmol/L (antenatal)^b^	8.63 (1.52)	8.57 (1.46)	8.69 (1.59)
GDM diagnosed (Weeks)^b^	25.3 (5.68)	24.8 (5.81)	25.7 (5.54)

Figures are numbers (percentages) unless otherwise specified. SD = Standard Deviation. HbA1c was not routinely collected at the study sites as GDM management in Hong Kong follows OGTT-based protocols.

a 1 USD = 7.78 HK.

b N = 242.

#### GAME intervention delivery and adherence

Fidelity to the intervention was high (see Table 2). Of 123 intervention participants, 105 (85%) were reached at the first telephone follow-up (median gestational age 37.6 weeks [IQR 37.3–38.0; range 35.6–39.1]) and 42 (34.1%) at the second. The most common reasons for not being reached were early delivery before scheduled call, inability to contact the participant after three attempts, and voluntary withdrawal from follow-up calls. Among those reached at the first follow-up, 90.1% (91/101) reported practicing AME. Among those practicing, 39.6% expressed milk twice or more daily, and 39.6% reported expressing colostrum for at least the recommended 10 min per session (i.e., met or exceeded the intended session duration). Adherence remained robust at the second follow-up (n = 41), where 90.2% of women continued to express colostrum, with 45.0% expressing for at least 10 min. Notably, 22 participants (21.6%) in the intervention group successfully collected and sent antenatal colostrum to the hospital for their infants use.

**Table 2:** The fidelity and compliance of the intervention group.

	First phone follow up n = 105 (85.4% of 123)	Second phone follow up n = 412 (34.1% of 123)
	n (%)	n (%)
Gestational age at telephone follow up (wk), mean (SD)	37.2 (0.2)	39 (0.5)
Self-reported practice of AME at home (yes/no)	91 (90.1)	40 (97.6)
Adherence to the GAME protocol (met all criteria: ≥2 times/day, ≥10 min/session)	61 (60.4)	37 (90.2)
Frequency of expression per day (n = 91)		
Less than twice	55 (60.4)	37 (92.5)
twice or more	36 (39.6)	3 (7.5)
Duration of expression each session (among those practicing) (n = 91)		
<10mins	55 (60.4)	22 (55)
≥10mins or more	36 (39.6)	18 (45)
Volume expressed		
No milk expressed	47 (51.6)	9 (22.5)
Milk expressed	44 (48.4)	31 (77.5)
Volume expressed in AME practice at home		
None	46 (51.1)	9 (22.5)
Visible drop (not measureable)	26 (28.9)	12 (30.0)
Scanty amount (not measureable)	18 (20.0)	13 (47.5)
Measured colostrum		
≥0.1 ml	12 (42.9)	4 (18.2)
≥0.5 ml	7 (25.0)	2 (9.1)
≥1 ml	3 (10.7)	3 (13.6)
≥2 ml	6 (21.4)	13 (59.1)
Collected colostrum in syringes, No. (%)	25 (27.2)	21 (52.5)
Stored colostrum at home, No. (%)	25 (27.2)	20 (50)
Recorded in log sheet	43 (47.3)	30 (75)
Stored expressed colostrum in home freezer	27 (100)	2 (4.8)
Attended additional breastfeeding clinic session	4 (4.0)	2 (4.8)

Total volume of antenatal milk expressed	Total intervention group (n = 123)
Range (ml)	0–23 ml
Mean (SD)	1.35 (3.61)
Antenatal breastmilk sent to hospital, n (%)	22 (10.2)
Overall adherence to GAME protocol, n = 105 (%)^a^	64 (61.0)

a Overall adherence calculated among all 105 participants reached at any telephone follow-up.

#### Primary outcome: exclusive breastfeeding

Using GLMM to account for repeated measures, the intervention group demonstrated significantly higher odds of exclusive breastfeeding over time compared to the control group (aOR = 2.43, 95% CI 1.17–5.04, p < 0.05) (see Table 3). In the time-point specific analysis, significant differences were observed at both discharge and 4 weeks postpartum. At discharge, the exclusive breastfeeding rate was significantly higher in the intervention group (16.3%) compared to the control group (8.9%) (aOR = 2.28, 95% CI 1.01–5.14, p < 0.05) (see Table 4). Similarly, at 4 weeks postpartum, the intervention group maintained a higher rate of exclusive breastfeeding (30.9%) than the control group (20.3%) (aOR = 2.00, 95% CI 1.07–3.74, p < 0.05). At 8 weeks postpartum, the rate remained numerically higher in the intervention group (29.3% vs 25.2%), but this difference did not reach statistical significance (aOR = 1.37, 95% CI 0.73–2.56).

**Table 3:** Generalised linear mixed-effects modelling of breastfeeding outcomes across time.

	Any breastfeeding		Exclusive breastfeeding	
	Model 1	Model 2	Model 3	Model 4
	aOR [95% CI]	aOR [95% CI]	aOR [95% CI]	aOR [95% CI]
Fixed effects				
GAME intervention				
AME group	0.68 [0.21–2.20]	0.38 [0.06–2.33]	2.43 ∗ [1.17–5.04]	4.00 ∗ [1.25–12.86]
Control group	Reference	Reference	Reference	Reference
Time				
Discharge	Reference	Reference	Reference	Reference
4 weeks postpartum	0.20 ∗∗∗ [0.08–0.45]	0.12 ∗∗∗ [0.03–0.40]	3.49 ∗∗∗ [1.89–6.44]	4.41 ∗∗ [1.65–11.79]
8 weeks postpartum	0.04 ∗∗∗ [0.01–0.10]	0.02 ∗∗∗ [0.00–0.08]	3.97 ∗∗∗ [2.15–7.35]	6.90 ∗∗∗ [2.57–18.50]
GAME × Time				
GAME × 4 wks		3.00 [0.59–15.13]		0.81 [0.24–2.76]
GAME × 4–8 wks		2.35 [0.43–12.85]		0.45 [0.13–1.53]
	**Variance (SD)**	**Variance (SD)**	**Variance (SD)**	**Variance (SD)**

Random effects				
(Intercept)	12.6	16.2 (4.03)	7 (2.65)	4.69 (2.17)
ICC	0.79	0.83	0.53	0.53
Observations	738	738	738	738
N	246	246	246	246
AIC	607.08	606.52	679.55	681.08
BIC	689.95	698.59	762.42	773.16
R2 (fixed)	0.20	0.21	0.17	0.19
R2 (total)	0.83	0.87	0.61	0.62

aOR = adjusted odds ratio. ICC = Intraclass Correlation Coefficient. AIC = Akaike Information Criterion. BIC = Bayesian Information Criterion. All continuous predictors are mean-centred and scaled by 1 standard deviation. All models adjusted for study site, maternal education, return to work postpartum, intention to breastfeeding, parity, and breastfeeding support. Models 1 and 3 (main effects) represent the pre-specified primary analyses for each outcome; Models 2 and 4 (with group-by-time interaction) are pre-specified secondary/exploratory analyses.

∗∗∗ p < 0.001; ∗∗ p < 0.01; ∗ p < 0.05.

**Table 4:** Breastfeeding outcomes across time.

	Total N = 246	Intervention N = 123	Control N = 123	χ^2^	p-value for χ^2^ test	aOR [95% CI]^a^
	n (%)	n (%)	n (%)			
Any Breastfeeding						
On discharge						
Yes	216 (87.8)	105 (85.4)	111 (90.2)	1.37	0.24	0.55 [0.24–1.27]
No	30 (12.2)	18 (14.6)	12 (9.8)			Reference
4 weeks postpartum						
Yes	195 (79.3)	98 (79.7)	97 (78.9)	0.02	0.87	1.11 [0.58–2.68]
No	51 (20.7)	25 (20.3)	26 (21.1)			Reference
8 weeks postpartum						
Yes	161 (65.4)	80 (65.0)	81 (65.9)	0.02	0.89	0.96 [0.55–1.69]
No	85 (34.6)	43 (35.0)	42 (34.1)			Reference
Exclusive breastfeeding						
On discharge						
Yes	31 (12.6)	20 (16.3)	11 (8.9)	2.99	0.08	2.28 [1.01–5.14] ∗
No	215 (87.4)	103 (83.7)	112 (91.1)			Reference
4 weeks postpartum						
Yes	63 (25.6)	38 (30.9)	25 (20.3)	3.61	0.06	2.00 [1.07–3.74]
No	183 (74.4)	85 (69.1)	98 (79.7)			Reference
8 weeks postpartum						
Yes	67 (27.2)	36 (29.3)	31 (25.2)	0.51	0.47	1.37 [0.73–2.56]
No	179 (72.8)	87 (70.7)	92 (74.8)			Reference

∗∗ p < 0.01; ∗ p < 0.05.

a Multivariable logistic regression adjusted for study site, maternal education, return to work postpartum, intention to breastfeed, parity, and breastfeeding support.

#### Co-primary outcome: any breastfeeding

Regarding co-primary outcome of any breastfeeding, there were no statistically significant differences between groups at any time point. Any breastfeeding rates were high in both groups at discharge (intervention 85.4% vs. control 90.2%), four weeks (79.7% vs 78.9%), and eight weeks (65.0% vs 65.9%) postpartum. The GLMM showed no overall intervention effect on any breastfeeding across the postpartum period (aOR = 0.68 [95% CI 0.21–2.20]).

#### Sensitivity and subgroup analyses

No significant differences in baseline characteristics were found between participants with complete (n = 210) and missing (n = 36) breastfeeding outcome data (all p > 0.1), supporting the MAR assumption (see Table S1). A complete-case sensitivity analysis, restricted to participants with non-missing data at all three time points, yielded a consistent result for exclusive breastfeeding (aOR = 3.06 [95% CI 1.25–7.51], p = 0.014), confirming that the primary findings are robust to the missing data handling approach (see Table S2). Exploratory subgroup analyses found no significant interaction between intervention effect and parity (interaction p = 0.37) or maternal education (interaction p = 0.28), although these analyses were underpowered for interaction testing (see Table S3).

#### Secondary outcomes: depressive symptoms and maternal wellbeing

The linear mixed-effects model revealed a significant intervention effect on maternal depressive symptoms over time. Specifically, there was a significant interaction between the intervention and time at 8 weeks postpartum (*β* = −1.43, 95% CI −2.58 to −0.28, p = 0.015). While no significant differences were observed at baseline or 4 weeks, at 8 weeks postpartum, women in the AME group reported significantly lower EPDS scores (mean 4.74, SE 0.77) compared to the control group (mean 6.48, SE 0.73). The estimated mean difference at this time point was −1.74 (95% CI −2.94 to −0.54, p = 0.004), indicating a clinically relevant reduction in depressive symptoms (see Table 5). Using the standard EPDS screening threshold of ≥ 10, the proportion of women screening positive was comparable at baseline (control 18·7% [23/123] vs intervention 22·0% [27/123]) and at 4 weeks (27·0% [24/89] vs 23·2% [19/82]). By 8 weeks, a significant difference had emerged: 19·5% of controls (15/77) screened positive compared with 5·7% of intervention participants (4/70) (p = 0·013). Among women with EPDS data at both baseline and 8 weeks, 6 control participants (7·8%) with sub-threshold baseline scores developed new-onset scores above the threshold, compared with 1 intervention participant (1·4%). Conversely, 12·9% of intervention participants with elevated baseline scores recovered to below the threshold, compared with 9·1% of controls (Table S4) (Table 6).

**Table 5:** Linear mixed-effects modelling on EPDS, BSES-SF and BSRQ.

	EPDS				BSES				BSRQ			
	Control	AME	Diff/***β***	p	Control	AME	Diff/***β***	p	Control	AME	Diff/***β***	p
	Mean (SE)	Mean (SE)	(95% CI)		Mean (SE)	Mean (SE)	(95% CI)		Mean (SE)	Mean (SE)	(95% CI)	
Marginal Means												
Baseline (T0)	6.83 (0.70)	6.52 (0.72)	−0.31 (−1.31, 0.68)	0.53	40.92 (1.86)	42.20 (1.93)	1.29 (−1.36, 3.94)	0.34	16.69 (0.59)	16.73 (0.61)	0.04 (−0.79, 0.87)	0.93
4 Weeks (T1)	7.41 (0.72)	6.68 (0.75)	−0.73 (−1.87, 0.40)	0.21	37.28 (1.92)	39.08 (2.01)	1.80 (−1.19, 4.80)	0.24	16.58 (0.60)	16.98 (0.63)	0.40 (−0.53, 1.33)	0.40
8 Weeks (T2)	6.48 (0.73)	4.74 (0.77)	−1.74 (−2.94, −0.54)	0.004	39.31 (1.95)	42.61 (2.06)	3.30 (0.12–6.48)	0.04	17.04 (0.61)	17.16 (0.64)	0.12 (−0.86, 1.11)	0.80
Fixed Effects												
GAME	–	–	−0.31 (−1.28, 0.65)	0.52	–	–	1.29 (−1.29, 3.87)	0.33	–	–	0.04 (−0.77, 0.85)	0.92
Time (4 weeks)	–	–	0.58 (−0.17, 1.34)	0.13	–	–	−3.64 (−5.63, −1.65)	<0.001	–	–	−0.11 (−0.71, 0.49)	0.73
Time (8 weeks)	–	–	−0.35 (−1.15, 0.44)	0.39	–	–	−1.61 (−3.70, 0.48)	0.13	–	–	0.35 (−0.28, 0.98)	0.28
GAME × 4 wks	–	–	−0.42 (−1.50, 0.67)	0.45	–	–	0.51 (−2.32, 3.35)	0.72	–	–	0.36 (−0.50, 1.21)	0.41
GAME × 8 wks	–	–	−1.43 (−2.58, −0.28)	0.02	–	–	2.01 (−1.01, 5.04)	0.19	–	–	0.08 (−0.83, 1.00)	0.86
Random Effects												
Intercept Var (SD)	–	–	7.58 (2.75)	–	–	–	55.40 (7.44)	–	–	–	5.71 (2.39)	–
ICC	–	–	0.363	–	–	–	0.347	–	–	–	0.351	–
Observation			564				569				576	
N			246				246				246	
AIC	–	–	4038.6	–	–	–	5444.8	–	–	–	3767	–
BIC	–	–	4135.3	–	–	–	5541.4	–	–	–	3863.7	–

Diff: marginal mean differences; EPDS: Edinburgh Postnatal Depression Scale; BSES-SF: Breastfeeding Self-Efficacy Scale–Short Form; BSRQ: Breastfeeding Self-Regulation Questionnaire. All models adjusted for study site, maternal education, return to work postpartum, intention to breastfeeding, parity, and breastfeeding support. EPDS baseline n = 246, 4 weeks n = 171 (control 89, intervention 82), 8 weeks n = 147 (control 77, intervention 70). BSES-SF baseline n = 246, 4 weeks n = 175, 8 weeks n = 148. BSRQ baseline n = 246, 4 weeks n = 179, 8 weeks n = 151.

**Table 6:** Infant and maternal postnatal health outcomes.

	Total	AME	Control	t test/χ2 test	p-value
	N = 246	N = 123	N = 123		
	Mean ± SD/n (%)	Mean ± SD/n (%)	Mean ± SD/n (%)		
Mode of delivery				2.16	0.35
Spontaneous Vaginal	164 (66.7)	81 (65.5)	83 (67.8)		
Instrumental Delivery	13 (5.4)	5 (3.9)	9 (7.0)		
Cesarean Section	69 (27.9)	38 (30.6)	31 (25.2)		
Maturity of delivery (wk)	38.74 (2.50)	38.58 (3.41)	38.90 (0.89)	−0.99	0.32
Baby birth weight (kg)	3.17 (0.39)	3.17 (0.43)	3.17 (0.35)	−0.06	0.95
Lowest neonatal blood glucose (Hemaistix in postnatal ward, mmol/L)	2.50 (1.79)	2.45 (1.80)	2.56 (1.78)	−0.49	0.63
Highest neonatal blood glucose (Hemastix in postnatal ward, mmol/L)	3.45 (2.50)	3.36 (2.50)	3.53 (2.49)	−0.49	0.63
Hypoglycemia in postnatal ward				1.5	0.22
No	197 (79.9)	94 (76.4)	103 (83.4)		
Yes	49 (20.1)	29 (23.6)	20 (16.6)		
Admission to NNU for hypoglycemia				0.59	0.44
No	216 (88.0)	106 (86.0)	111 (89.9)		
Yes	30 (12.0)	17 (14.0)	12 (10.1)		
Admission to NNU for other reasons				0.18	0.67
No	176 (71.7)	87 (70.4)	90 (73.0)		
Yes	70 (28.3)	36 (29.6)	33 (27.0)		
Length of NNU stay, d (n = 83) (among admitted neonates)	4.07 (3.07)	4.14 (3.33)	4.00 (2.80)	0.21	0.84
Baby decreased body weight in postnatal ward					
No	202 (82.0)	99 (80.8)	102 (83.3)	0.12	0.73
Yes	44 (18.0)	24 (19.2)	21 (16.7)		
Lactation consultant in postnatal ward					
No	164 (66.8)	79 (64.4)	85 (69.3)	0.29	0.59
Yes	82 (33.2)	44 (35.6)	38 (30.7)		
OGTT (fasting glucose) at 6 wk postpartum, mmol/L (n = 42)	4.96 (0.46)	5.07 (0.49)	4.88 (0.43)	1.30	0.20
OGTT (2-h glucose) at 6 wk postpartum, mmol/L (n = 42)	6.68 (2.05)	6.77 (2.33)	6.62 (1.86)	0.24	0.14

NNU, Neonatal unit; OGTT, oral glucose tolerance test. Length of NNU stay is reported among attendees only (42 of 221 women attended the 6-week OGTT; intervention = 18, control n = 24). Hemastix refers to point-of-care capillary blood glucose testing used in Hong Kong neonatal wards.

For breastfeeding self-efficacy, a significant group difference emerged by the end of the study period. Although the overall interaction term for the intervention was not statistically significant, pairwise comparisons of the estimated marginal means showed that at 8 weeks postpartum, the AME group had significantly higher breastfeeding self-efficacy scores (mean 42.61, SE 2.06) than the control group (mean 39.31, SE 1.95). The mean difference was 3.30 (95% CI 0.12–6.48, p = 0.042). A significant main effect of time was observed at 4 weeks (*β* = −3.64, p < 0.001), indicating a general decline in self-efficacy scores for both groups shortly after birth before stabilising. No significant differences were observed in breastfeeding self-regulation scores between the intervention and control groups at any time point (p > 0.05). The LMM analysis indicated no significant main effects of the intervention or interaction effects over time.

#### Maternal and neonatal safety

There were no significant differences between the groups regarding maternal or neonatal safety outcomes. The mode of delivery (spontaneous vaginal, instrumental, or cesarean section) was similar between groups (p = 0.348). Importantly, the intervention did not increase the risk of neonatal complications; rates of admission to the neonatal unit (NNU) were comparable between the intervention (29.6%) and control groups (27.0%) (p = 0.672). Additionally, the incidence of neonatal hypoglycemia did not differ significantly between groups (23.6% vs 16.6%, p = 0.221).

## Discussion

This two-site, assessor-blinded randomised controlled trial is, to our knowledge, the first randomized controlled trial in Hong Kong to evaluate the effectiveness, feasibility, and safety of a structured antenatal milk expression programme among women with GDM. Our principal finding is that the GAME intervention significantly increased the rate of exclusive breastfeeding at 4 weeks postpartum and provided a robust protective effect against maternal depressive symptoms at 8 weeks postpartum. Importantly, the intervention was safe, with no increase in adverse maternal or neonatal outcomes compared to standard care.

The GAME intervention demonstrated a significant improvement in exclusive breastfeeding rates. The intervention group showed 2.69 times higher odds of exclusive breastfeeding across the postpartum period compared to standard care. This effect was most pronounced at 4 weeks postpartum, where the intervention group achieved a 14.2 percentage point absolute increase in exclusive breastfeeding rates. While exclusive breastfeeding rates declined in both groups by 8 weeks postpartum, the intervention group maintained numerically higher rates throughout the follow-up period. This decline is consistent with broader population trends documented globally.^20^ Importantly, the intervention did not significantly impact any breastfeeding rates, suggesting that AME primarily influenced breastfeeding exclusivity rather than initiation, which was already high in both groups.

Perhaps most striking was the intervention's substantial protective effect against postpartum depressive symptoms. At 8 weeks postpartum, women in the intervention group reported EPDS scores that were 1.71 points lower than controls. The clinical relevance of this difference is supported by threshold analysis. At 8 weeks, only 5.7% (6/106) of intervention participants screened positive for probable depression (EPDS ≥10) compared with 19.5% (17/87) of controls (p = 0.013), corresponding to an absolute risk reduction of 13.8 percentage points and a number needed to treat of 7. The standardised effect size (Cohen's d = 0.60) represents a medium effect. These findings indicate that the observed mean difference translates into a meaningful reduction in the proportion of women at risk of postpartum depression, not merely a statistically significant but clinically trivial change. This finding is particularly noteworthy given that women with GDM are recognised to be at elevated risk for postpartum depression.^21^ Proposed mechanisms by which the intervention may confer mental health benefit include enhanced maternal confidence through skill acquisition and increased breastfeeding self-efficacy,^22^ reduced anxiety about infant feeding through hands-on preparation,^23^ and the established association between successful breastfeeding establishment and lower postpartum depression^24^; these pathways are biologically and psychologically plausible but warrant confirmation in future mediation analyses. The empowering effect of proactive self-management during pregnancy may contribute additional benefits.

The safety profile of the GAME intervention was reassuring across all measured parameters. Contrary to theoretical concerns about stimulating preterm labour, there were no significant differences in gestational age at delivery, mode of delivery, or maternal complications. Crucially, neonatal outcomes, including rates of hypoglycaemia requiring neonatal unit admission, were comparable between groups. This finding is important because a primary rationale for AME is to provide an alternative to formula supplementation for managing neonatal hypoglycaemia, a common complication in infants born to mothers with GDM. Although 21.6% of intervention participants successfully brought expressed colostrum to the hospital, the lack of difference in neonatal hypoglycaemia rates requires consideration. This suggests either that the volume of antenatal colostrum collected was insufficient to prevent hypoglycaemia, or that current hospital protocols for managing neonatal blood glucose levels operate independently of colostrum availability. Nevertheless, the availability of expressed colostrum may have facilitated exclusive breastfeeding by avoiding formula introduction during the critical early postpartum period, thereby supporting longer-term breastfeeding exclusivity.^25^ We note that this trial was powered for the primary breastfeeding outcome, and the sample size provides limited statistical power to detect modest differences in rare safety events such as neonatal hypoglycaemia or NNU admission; thus, we cannot exclude small or rare adverse effects that would be detectable in a larger cohort. Additionally, rare or measured outcomes (e.g. transient neonatal hypoglycaemia not requiring admission, maternal nipple trauma, or delayed lactogenesis) were not captured. Safety findings should therefore be interpreted as preliminary and require confirmation in larger multicentre studies.

Our findings align with and extend the limited existing evidence on AME. Previous trials have produced mixed results regarding breastfeeding outcomes. The landmark 2017 Australian DAME trial by Förster and colleagues, which included women with diabetes (both GDM and pre-existing diabetes), found that AME did not significantly improve breastfeeding rates at hospital discharge or 6 months postpartum.^10^ However, the authors noted potential benefits in specific subgroups. Several key differences between the DAME trial and our study merit consideration when interpreting these divergent findings. First, the DAME trial employed a cluster-randomised design across six hospitals, whereas our study used individual-level randomisation, which may reduce contamination and enhance intervention fidelity. Second, the DAME trial included women with pre-existing diabetes (type 1 and type 2) in addition to GDM, whereas our sample was restricted to women with GDM, who may differ in baseline breastfeeding intentions, metabolic profiles, and psychological readiness. Third, the DAME intervention consisted primarily of a single education session with written materials, whereas our GAME programme included a structured 45-min face-to-face session supplemented by weekly telephone support, providing ongoing reinforcement of skills and motivation. Fourth, the cultural context differs substantially: our study was conducted in Hong Kong, where AME is not routine practice and where culturally tailored, trust-based delivery by known midwives may have enhanced uptake and adherence. In contrast, smaller studies and retrospective analyses conducted in other settings have also reported improvements in initiation and continuation of breastfeeding, breastfeeding self-efficacy, and reductions in formula supplementation.^26^^,^^27^ Taken together, these comparisons suggest that the positive findings in our trial may be attributable to a combination of the GDM-specific focus, the multi-component and culturally adapted intervention design, and the provision of ongoing telephone support to reinforce adherence.

The reduction in depressive symptoms observed in our study is particularly novel. This outcome has not been consistently reported in previous AME trials. This may reflect differences in study populations, intervention intensity, or outcome measurement. The psychological benefits of AME may be especially salient in the GDM population. Women with GDM often experience heightened anxiety about pregnancy complications and infant health.^28^ By providing a concrete, actionable strategy to support infant feeding and health, AME may help restore a sense of agency and control during a vulnerable period.

Adherence to the intervention was high among those contacted: at the first telephone follow-up (response rate 96.0% [101/105]), 90.1% (91/101) of participants reported practicing AME, and at the second follow-up (response rate 93.2% [41/44] of continuing participants), 90.2% (37/41) reported continued practice. These adherence estimates are based on a subset of respondents and may be subject to self-selection bias, as women who were more motivated to practice AME may also have been more likely to respond to follow-up calls; thus, true adherence in the full intervention group may be somewhat lower. Nevertheless, the fact that high adherence was reported even in a setting where AME is not routine—Hong Kong, where cultural conservatism around pregnancy practices is common—suggests that, when delivered by trusted healthcare providers using culturally appropriate, evidence-based communication, AME can be acceptable and feasible. The collection of antenatal colostrum for infant use (21.6% of those who delivered at study hospitals) further supports the intervention's feasibility in this context. Among women who expressed colostrum, most reported small yields (<1–2 ml per session), consistent with typical antenatal colostrum volume. Although some women brought expressed colostrum to the labour ward, its actual administration was not systematically recorded, and in some cases, colostrum was not used—either because neonatal hypoglycemia did not occur or because standard hospital protocols prioritised immediate breastfeeding or formula supplementation. Thus, low utilisation may reflect a combination of limited expressed volume, maternal hesitation, and institutional practices rather than an inherent flaw in the intervention. Whether colostrum volume or actual in-hospital utilisation moderates breastfeeding outcomes was not assessed in this study but merits evaluation in future trials with embedded process evaluations.

Women with GDM face distinct physiological barriers to establishing exclusive breastfeeding. A 2016 study found that factors associated with higher breastfeeding self-efficacy among women with GDM included earlier initiation of breastfeeding and no perceived delayed lactogenesis II.^29^ Women with GDM are more likely to experience delayed lactogenesis II, and their infants are prone to neonatal hypoglycaemia.^30^^,^^31^ These biological challenges are compounded by psychological factors. Women with GDM report less breastfeeding self-efficacy and require more support compared to those without GDM.^32^^,^^33^ Our intervention addressed these challenges through education, skill-building, and ongoing support.

This study has several notable strengths. The randomised controlled design with assessor blinding minimises bias in outcome assessment. The inclusion of both efficacy and safety outcomes provides a comprehensive evaluation of the intervention's risk–benefit profile. The use of validated, culturally appropriate measurement tools enhances the reliability and relevance of our findings. The relatively large sample size for an intervention trial in this population strengthens our conclusions. The inclusion of participants from two sites enhances the generalisability of our results within the Hong Kong context.

However, several limitations warrant consideration. First, the loss to follow-up due to participants delivering at non-study hospitals (8.5% overall) may introduce potential selection bias. However, baseline characteristics were balanced between groups. Second, the inability to blind participants and care providers to group allocation may have introduced performance bias, particularly for self-reported outcomes such as depressive symptoms and breastfeeding self-efficacy. Third, the relatively short follow-up period of eight weeks limits our ability to assess longer-term breastfeeding outcomes and the sustained impact on maternal mental health. No additional follow-up data beyond eight weeks were collected for this cohort. A follow-up study assessing breastfeeding and metabolic outcomes at six and twelve months is planned. Given that the metabolic benefits of breastfeeding for diabetes prevention accrue with longer duration,^34^ future studies should examine whether early improvements in exclusive breastfeeding translate into sustained breastfeeding beyond 6 months. Fourth, we did not collect detailed data on the volume of colostrum expressed antenatally or actually used postpartum, limiting our ability to establish a dose-response relationship. Finally, our study was conducted in a specific cultural context with particular healthcare system characteristics. All participants were Chinese women in Hong Kong, and findings may limit generalisability to other ethnic populations and healthcare settings. Additionally, participants were volunteers and may have been more motivated to breastfeed than the wider GDM population, potentially limiting representativeness even within the local context. Baseline breastfeeding self-efficacy was assessed during pregnancy (36 weeks' gestation) using the Breastfeeding Self-Efficacy Scale (BSES), which was originally developed and validated for postpartum use with currently breastfeeding women. Although the BSES has been used prenatally in prior studies to assess antenatal breastfeeding confidence, its psychometric properties in this context have not been formally established. A validated prenatal instrument—the Prenatal Breastfeeding Self-Efficacy Scale (PBSES)^35^ —is available and should be considered in future trials. This limitation should be considered when interpreting baseline and longitudinal BSES scores in our study.

The findings of this trial have important implications for clinical practice and public health policy. For clinicians caring for women with GDM, our results suggest that offering a structured AME programme represents a safe, feasible, and effective strategy to enhance exclusive breastfeeding and support maternal mental health. The intervention requires minimal resources: a single education session, provision of collection supplies, and two follow-up telephone calls. This makes the intervention potentially cost-effective and scalable within existing antenatal care services.

For health policymakers, our findings support the integration of AME education into routine diabetes in pregnancy care pathways. Current clinical guidelines on AME remain inconsistent, partly due to limited high-quality evidence, particularly from Asian populations. Our trial contributes robust evidence that can inform guideline development and revision. Moreover, given the established link between breastfeeding duration and reduced diabetes risk in mothers with a history of GDM, interventions that improve exclusive breastfeeding may contribute to diabetes prevention at a population level.

The mental health benefits observed in our study also warrant attention. Postpartum depression screening and intervention are increasingly recognised as essential components of maternal healthcare. Yet effective preventive strategies remain limited. Our findings suggest that AME, delivered as part of routine antenatal care, may serve a dual purpose: supporting infant feeding while simultaneously promoting maternal psychological wellbeing. This aligns with contemporary models of perinatal care that emphasis holistic, woman-centred approaches.

However, successful implementation will require careful attention to several factors. Healthcare providers need adequate training in AME techniques and counselling to deliver the intervention confidently and consistently. Hospital protocols may need revision to ensure that expressed colostrum brought to the hospital is appropriately stored, labelled, and utilised. Cultural beliefs and concerns about AME must be respectfully addressed through culturally sensitive educational materials and messaging. Finally, ongoing quality improvement efforts should monitor both implementation fidelity and outcomes to ensure that real-world effectiveness matches trial efficacy.

Several important questions remain that should be addressed in future research. First, longer-term follow-up studies are needed to determine whether the improvements in exclusive breastfeeding at 4–8 weeks translate into sustained breastfeeding duration at 6 months and beyond. Second, economic evaluations would help establish the cost-effectiveness of AME programmes from both healthcare system and societal perspectives. Third, studies examining the optimal timing, frequency, and duration of antenatal expression would refine intervention protocols. Fourth, research exploring the mechanisms underlying the mental health benefits would enhance our understanding. Studies should investigate whether benefits are mediated through breastfeeding success, self-efficacy, or other pathways. This could potentially identify additional intervention targets. Fifth, trials in diverse populations and healthcare settings are needed to assess generalisability and identify population-specific adaptations. Finally, given the heterogeneity within the GDM population, future studies might explore whether AME is particularly beneficial for specific subgroups, such as women requiring insulin therapy or those with additional risk factors for breastfeeding difficulties.

In this randomised controlled trial, a structured antenatal milk expression programme significantly improves exclusive breastfeeding rates and reduces postpartum depressive symptoms among Chinese women with GDM. The intervention did not result in adverse maternal or neonatal safety outcomes. These findings support the integration of AME into routine antenatal care for women with GDM as a safe, feasible, and effective strategy to promote both infant and maternal health. Given the substantial long-term health implications of GDM for both mothers and their offspring, interventions that support breastfeeding and maternal wellbeing represent important opportunities for diabetes prevention and the promotion of lifelong health. Further research with longer follow-up and in diverse populations will help optimise implementation and maximise the public health impact of this promising intervention.

## Contributors

YNL and KYWL conceived and designed the study, and drafted the original manuscript. CCOL and WCL supervised participant recruitment at the study sites. YNL recruited participants and collected data. YNL, KWYL and YD performed the statistical analysis, accessed and verified the underlying data. PHC, YD, and KYWL critically revised the manuscript for important intellectual content. KYWL and PHC supervised the project, and KYWL and YNL obtained funding. All authors critically reviewed the manuscript, provided intellectual input, and approved the final version for submission.

## Data sharing statement

Data collected for the study, including deidentified individual participant data and a data dictionary defining each field in the set, can be made available to corresponding author on reasonable request and after signing appropriate data sharing agreements and must be approved by the relevant ethics committees and data custodians.

## Declaration of interests

All authors declare no competing interests.

## Appendix A Supplementary data

Protocol

## References

[1] Magliano D., Boyko E., IDF Diabetes Atlas 11th edition scientific committee IDF DIABETES ATLAS. 11th ed. Brussels: International Diabetes Federation. https://www.ncbi.nlm.nih.gov/books/NBK581934/.

[2] Bellamy L., Casas J.P., Hingorani A.D., Williams D. (2009). Type 2 diabetes mellitus after gestational diabetes: a systematic review and meta-analysis. Lancet.

[3] Oladimeji O.I., Ohene-Agyei P., Liu Q. (2025). In utero exposure to gestational diabetes and child health at age three to seven: a cohort study. J Pediatr.

[4] Gunderson E.P., Hurston S.R., Ning X. (2015). Lactation and progression to type 2 diabetes mellitus after gestational diabetes mellitus: a prospective cohort study. Ann Intern Med.

[5] Victora C.G., Bahl R., Barros A.J. (2016). Breastfeeding in the 21st century: epidemiology, mechanisms, and lifelong effect. Lancet.

[6] Aune D., Norat T., Romundstad P., Vatten L.J. (2014). Breastfeeding and the maternal risk of type 2 diabetes: a systematic review and dose-response meta-analysis of cohort studies. Nutr Metab Cardiovasc Dis.

[7] Chapman D.J., Pérez-Escamilla R. (1999). Identification of risk factors for delayed onset of lactation. J Am Diet Assoc.

[8] Björvang R.D., Liakea I., Carpentsier B., Kozinszky Z., Skalkidou A., Fransson E. (2024). Association of diabetes mellitus in pregnancy and perinatal depression. Psychosom Med.

[9] Forster D.A., Jacobs S., Amir L.H. (2014). Safety and efficacy of antenatal milk expressing for women with diabetes in pregnancy: protocol for a randomised controlled trial. BMJ Open.

[10] Forster D.A., Moorhead A.M., Jacobs S.E. (2017). Advising women with diabetes in pregnancy to express breastmilk in late pregnancy (Diabetes and Antenatal Milk Expressing [DAME]): a multicentre, unblinded, randomised controlled trial. Lancet.

[11] Liao S., Mei J., Song W. (2014). The impact of the International Association of Diabetes and Pregnancy Study Groups (IADPSG) fasting glucose diagnostic criterion on the prevalence and outcomes of gestational diabetes mellitus in Han Chinese women. Diabet Med.

[12] Wight N.E., Med A.B. (2021). ABM clinical protocol #1: guidelines for glucose monitoring and treatment of hypoglycemia in term and late preterm neonates, revised 2021. Breastfeed Med.

[13] World Health Organization Infant and young child feeding. https://www.who.int/westernpacific/health-topics/infant-nutrition.

[14] Ip W.Y., Yeung L.S., Choi K.C., Chair S.Y., Dennis C.L. (2012). Translation and validation of the Hong Kong Chinese version of the breastfeeding self-efficacy scale-short form. Res Nurs Health.

[15] Lau C.Y.K., Fong D.Y.T., Chan V.H.S. (2022). The effect of maternal self-regulated motivation on breastfeeding continuation. Matern Child Hlth J.

[16] Lau C.Y.K., Fong D.Y.T., Choi A.Y.Y., Ng J.W.Y., Sing C., Tarrant M. (2017). Development and measurement properties of the Chinese breastfeeding self-regulation questionnaire. Midwifery.

[17] Lee D.T., Yip S.K., Chiu H.F. (1998). Detecting postnatal depression in Chinese women. Validation of the Chinese version of the Edinburgh Postnatal Depression Scale. Br J Psychiatry.

[18] Mattar C.N., Chong Y.S., Chan Y.S. (2007). Simple antenatal preparation to improve breastfeeding practice: a randomized controlled trial. Obstet Gynecol.

[19] Imdad A., Yakoob M.Y., Bhutta Z.A. (2011). Effect of breastfeeding promotion interventions on breastfeeding rates, with special focus on developing countries. BMC Public Health.

[20] Shahrani A.S.A., Hushan H.M., Binjamaan N.K., Binhuwaimel W.A., Alotaibi J.J., Alrasheed L.A. (2021). Factors associated with early cessation of exclusive breast feeding among Saudi mothers: a prospective observational study. J Family Med Prim Care.

[21] Wilson C.A., Newham J., Rankin J. (2020). Is there an increased risk of perinatal mental disorder in women with gestational diabetes? A systematic review and meta-analysis. Diabet Med.

[22] Vieira E.S., Caldeira N.T., Eugenio D.S., Lucca M.M.D., Silva I.A. (2018). Breastfeeding self-efficacy and postpartum depression: a cohort study. Rev Lat Am Enfermagem.

[23] Agler R.A., Zivich P.N., Kawende B., Behets F., Yotebieng M. (2021). Postpartum depressive symptoms following implementation of the 10 steps to successful breastfeeding program in Kinshasa, Democratic Republic of Congo: a cohort study. PLoS Med.

[24] Xia M., Luo J., Wang J., Liang Y. (2022). Association between breastfeeding and postpartum depression: a meta-analysis. J Affect Disord.

[25] Chantry C.J., Dewey K.G., Peerson J.M., Wagner E.A., Nommsen-Rivers L.A. (2014). In-hospital formula use increases early breastfeeding cessation among first-time mothers intending to exclusively breastfeed. J Pediatr.

[26] Casey J.R.R., Banks J., Braniff K., Buettner P., Heal C. (2019). The effects of expressing antenatal colostrum in women with diabetes in pregnancy: a retrospective cohort study. Aust N Z J Obstet Gynaecol.

[27] Al Bekai E., Beaini C.E., Kalout K. (2025). The hidden impact of gestational diabetes: unveiling offspring complications and long-term effects. Life (Basel).

[28] OuYang H., Chen B., Abdulrahman A.M., Li L., Wu N. (2021). Associations between gestational diabetes and anxiety or depression: a systematic review. J Diabetes Res.

[29] Chertok I.R., Sherby E. (2016). Breastfeeding self-efficacy of women with and without gestational diabetes. MCN Am J Matern Child Nurs.

[30] McGuinness D., Daniel U., Brien D.O. (2025). Facilitators and barriers to establishing lactation among women with diabetes?. Diabet Med.

[31] Gunderson E.P., Crites Y., Chiang V. (2012). Influence of breastfeeding during the postpartum oral glucose tolerance test on plasma glucose and insulin. Obstet Gynecol.

[32] Otter G., Davis D., Kurz E. (2024). Promoting breastfeeding in women with gestational diabetes mellitus in high-income settings: an integrative review. Int Breastfeed J.

[33] Qian P., Duan L., Lin R. (2022). How breastfeeding behavior develops in women with gestational diabetes mellitus: a qualitative study based on health belief model in China. Front Endocrinol (Lausanne).

[34] Pinho-Gomes A.C., Morelli G., Jones A., Woodward M. (2021). Association of lactation with maternal risk of type 2 diabetes: a systematic review and meta-analysis of observational studies. Diabetes Obes Metab.

[35] Wells K.J., Thompson N.J., Kloeblen-Tarver A.S. (2006). Development and psychometric testing of the prenatal breast-feeding self-efficacy scale. Am J Health Behav.

