# Supplementary material for: The Gestational Diabetes Antenatal Milk Expression (GAME) trial: effects on breastfeeding outcomes and maternal mental health—A randomised controlled trial
Source: eClinicalMedicine. 2026 Aug 5;98:104130. doi: 10.1016/j.eclinm.2026.104130 (PMC13463982; doi:10.1016/j.eclinm.2026.104130)
Supplement: Supplementary file 1 [file mmc1.docx]

# 1 General Information

Date: 2^nd^ Dec 2021

Title: The Effects and Experience of a Gestational Diabetes and Antenatal Human Milk Expression (GAME) Programme in Hong Kong Chinese Women.

Authorised to sign-off protocol: Dr Kris Lok and Dr Patsy Chau

Academic Supervisor: Dr Kris Lok, Assistant Professor, School of Nursing, The University of Hong Kong

Investigators:

| **Name** | **Relevant qualification** | **Department/ Institution/Hospital** |
| --- | --- | --- |
| Ms Leung Yu Ngai | FHKAN (Midwifery), MPH, IBCLC, RNM, RN | Department of Obstetrics and Gynaecology, KWH |
| Dr Patsy Chau | PhD | School of Nursing, The University of Hong Kong |
| Dr Leung Wing Cheong | MBBS, MRCOG, FHKAM, FHKCOG, Cert RCOG (Maternal and Fetal Med), FRCOG, MD | Department of Obstetrics and Gynaecology, KWH |
| Ms Christine Lam | Master of Midwifery, Fellow member of HK college of Midwives, IBCLC, RNM, RN | Department of Obstetrics and Gynaecology, QEH |

Addresses

1 Dept. of Obstetrics and Gynaecology, Kwong Wah Hospital, 25 Waterloo Road, Kowloon, Hong Kong

2 School of Nursing, 4/F William M. W. Mong Block, Faculty of Medicine, 21 Sassoon Road, Hong Kong

Project sites:

1. Dept. of Obstetrics and Gynaecology, Kwong Wah Hospital, 25 Waterloo Road, Kowloon, Hong Kong; and
2. Dept. of Obstetrics and Gynaecology, Queen Elizabeth Hospital, 30 Gascoigne Road, Kowloon, Hong Kong

# Background Information

Diabetes is a common chronic disease. The International Diabetes Federation (IDF),^1-3^ it is estimated to be 9.3% has diabetes and were projected to raise to 10.9% by 2045, with rising incidence of obesity, unhealthy diet, and physical inactivity. The global prevalence of impaired glucose tolerance is estimated to 7.5% and projected to reach 8.6% by 2045.The global prevalence of diabetes among adults doubled in recent decades. It is the ninth leading cause of death globally in 2019.^4^ In 2017, it was estimated USD 727 billions of global health expenditure on diabetes.^3^ It is an emerged global public health concern. According to the report of IDF Diabetes Atlas 9th edition,^2^ China accounted to top 10 countries for numbers of people living with diabetes in 2019 and is estimated 11% prevalence of Diabetes in Hong Kong. The prevalence of diabetes nearly doubled in the past few decades. It is estimated to a million of diabetic adults by 2030 in Hong Kong which increase economic and healthcare system burden.

Gestational Diabetes Mellitus (GDM) is defined as a type of diabetes first diagnosed in the second or third trimester of pregnancy.^5-6^ The incidence of GDM has been increasing worldwide and varied globally. It affected 16% (1 in 6) of hyperglycaemia in pregnancy.^2^ GDM accounts for 86% of hyperglycaemia in pregnancy. Overall, women with previous GDM have a 7-fold higher risk of developing DM over their lifetime.^7^ A cohort study conducted in Hong Kong, it shows that women with persistent postpartum impaired glucose tolerance after GDM have a high risk of developing diabetes mellitus.^8^ It has emerged global public health concern.

Exclusive breastfeeding is global recognized as the optimal infant feeding. It is especially strongly recommended and supported to the maternal history of GDM.^9-12^Women with GDM breastfeed her baby, improve lipid and glucose metabolic profiles during the first 3 months following delivery.^12-15^ Lactation has an insulin-sparing effect on the mother and improves insulin sensitivity and glucose metabolism.^16-17^ There is apparent evidence that it prevents metabolic syndrome, preserve beta cell function, improved weight loss and reduced obesity. Empirical literature demonstrated that breastfeeding and maternal risk of Type 2 Diabetes (T2D) in positive dose-response relationship.^18-24^ Studies found that breastfeeding for > 3 months decrease in the risk of T2DM by 40%.^24^ Evidence shown longer lifetime duration of lactation is associated with T2D and a favorable glucose metabolic biomarker profile.^25^ High intensity of breastfeeding significantly improves postpartum glucose metabolism and long-term reduction in cardiometabolic disease.^20^

However, exclusive breastfeeding rate for 6 months is still low in Hong Kong and worldwide.^26-28^ Globally, only 38% of infants aged ≤ 6 months are exclusively breastfed vs. 26.3% in Hong Kong. Evidence shows that GDM women have breastfeeding challenges and barriers.^11,29-32^ It also negatively associated with initiation of exclusive breastfeeding and breastfeeding duration.^11,30-31,33-34^ Women with diabetes experience delay in lactogenesis and infant have increased risk hypoglycaemia, they require blood monitoring and are often admit to the neonatal unit. If infants are hypoglycaemic and their mother is unable to express breastfeeding and breastfeeding, they may receive formula supplementation or intravenous glucose.^35^ Diabetes women may cause temporary insulin imbalance required glucose and initiation of lactation. They are more likely to have early receiving infant of formula. Besides, evidence indicated that low maternal confidence, increase depressive symptoms and breastfeeding self-efficacy among GDM women.^11,36^ Therefore, early lactation support was necessary.^32,37^ Greater support for GDM women reduces the risks of hypoglycaemia, formula supplementation and early weaning.^38^ It has been proposed that hand expression of breast milk in pregnancy may promote breastfeeding. Mothers with diabetes can express colostrum antenatally for use rather than infant formula if necessary.^39-42^

**Literature Review and Knowledge Gaps**

Growing evidence and gained popularity of Antenatal Milk Expression (AME) in overseas countries. It appears to be an acceptable intervention. They advise women at risk of early lactation problems.^37, 38^ However, these recommend theoretical practices have not well recognised and very limited evidence regarding the safety and efficacy of the practice.^39,43-47^ Previous studies reported conflicting concerns on stimulate uterine contraction, increase admission to neonatal unit.^48^ However, recent critical review concluded that substantial benefits of early feeding of colostrum outweigh the lack of evidence associated with risk of preterm labour.^40,46^

Very limited evidence (only two studies) in clinical trial^39^ and a systematic review from Cochrane review investigated the AME for diabetes women for improving infant outcomes.^35^ A large multicentre, unblinded randomised controlled trial in Australia showed that low-risk women with diabetes during pregnancy can express colostrum without causing harm or risk of infant hypoglycaemia from 36 weeks of gestation.^35,39,49^ It found increase in exclusive breastfed in the first 24 hours for primigravida women. However, the recommended practices have not well recognized, and very limited evidence stated the safety and efficacy of the practice. Previous studies reported conflicting concerns on stimulate uterine contraction and increase admission to neonatal unit. However, recent critical review concluded that substantial benefits of early breastmilk prevail no evidence associated with risk of preterm labour.^46^ Evidence found that AME improves postnatal lactation performance and confidence, influence neonatal unit admission, increase exclusive breastfeeding and reduce neonatal hypoglycemia.^43-44,50-51^ Few studies found that AME increased confidence and contributed to better preparation on breastfeeding.^44,51-52^ To date, AME on depressive symptoms is under examined.

AME is not widely practice with inconsistent advice and numbers of practice are unknown in Hong Kong. It may raise concern on culture diversity regarding conservative culture in Chinese. Studies identified negative feelings of embarrassment, difficulties with expression and physical pain experienced with the inadequate advice about the benefits of collecting colostrum.^42,44,51-53^ To my knowledge, there is lack of local article or evidence on the feasibility and acceptability of AME practice for GDM women in Hong Kong. There is lack of local clinical protocol on the implementation of AME and instructions on the practice. There is lack of consensus in protocols and inconsistent advice on the practices in worldwide. There is very limited existing evidence in sociopsychological approaches on the AME practice in pregnancy women. The local practice and experience of women in Hong Kong are not explored and not understand. Given cultural differences in breastfeeding motivation and self- regulation as compared with those of oversea countries.^54^ The motivation of Hong Kong pregnant women engages in antenatal education and receiving social and breastfeeding support may be differ from women in the West. Thus, it is important to evaluate the efficacy and experience of the AME on Hong Kong Chinese to assess cultural acceptability and effectiveness in supporting baby-friendly hospital practice and breastfeeding continuation.

Breastfeeding confidence and breastfeeding behaviour are strong predictors of breastfeeding exclusivity and duration.^55-58^ Breastfeeding self-efficacy theory developed based on Bandura’s social cognitive theory which provide theoretical framework which guide the evaluation of interventions.^59-60^ Studies show the effective intervention based on the self-efficacy theory in enhancing mothers’ breastfeeding self-efficacy and the breastfeeding rates. Theoretical framework on breastfeeding education using self-efficacy theory has been showed to correlated with breastfeeding outcomes. Limited qualitative study^36^ compares breastfeeding self-efficacy on GDM women and positive impacts on the self-efficacy on breastfeeding prevalence and duration.^55-56^ One study found perinatal individual theory based breastfeeding education had positive effects on breastfeeding self-efficacy and breastfeeding rate of women with GDM.^61^

There is very limited qualitative study on the experiences and perceptions on the gestational diabetes on breastfeeding. Very rare qualitative studies on the experience on the collection and storage of AME are unexplored. The phenomenon on antenatal milk expression is published in two qualitative studies^44,53^ and two mixed studies in western countries.^51-52^ A few studies revealed midwives teach the skill AME, but their advice given to women varied substantially.^45^ The possible facilitators and challenges on the experiences on collection, storage and use of expressed milk are little known. Studies identified negative feelings of embarrassment, difficulties with expression, experienced physical pain and inadequate advice about the benefits of collecting colostrum antenatally.^44^ It also explored hand fatigue and breast soreness associated with the process. It discourages the intention of practice, limited AME frequency and storing colostrum.^44,51^ Besides, the barriers to use, privacy, milk collection and storage impact the use of antenatal expressed milk.^51^

# 2 Objectives and Purpose

**Purpose of the Study**

1.	To determine the effectiveness of antenatal human milk expression on exclusive breastfeeding and breastfeeding self-efficacy and depressive symptom on GDM women

2.	To follow up the previous research and help to explain the causal theories or models in the future.

3.	To understand experience of AME on breastfeeding’s intention and personal beliefs which affect their behaviour.

4.	The embedded meaning of the experience can make sense of the acceptability and feasibility of the AME practice.

5.	Identify the barriers and facilitators of the AME practice suggesting for improvements to tailor the interventions

The objectives of this study are to examine (1) the acceptability and feasibility of AME as breastfeeding intervention to improve breastfeeding for GDM women, how and why AME works or does not work using qualitative research (hence, no hypothesis); (2) to understand the experience and perceptions of antenatal milk expression among the GDM women and (3) to investigate the efficacy of AME practice on the breastfeeding exclusivity. The secondary aims are determining whether AME as mediating variables in the effect on the breastfeeding self-efficacy among Hong Kong GDM women in Hong Kong.

The study hypotheses are (1) Antenatal human milk expression (AME) will increase breastfeeding exclusivity on hospital discharge, at 4 weeks and 8 weeks postpartum compare women receiving standard care (2) AME will improve the breastfeeding self-efficacy and reduce depressive symptoms of women after birth.

# 3 Design

**Design and Methods**

Women diagnosed with GDM in the index pregnancy will be recruited after the diagnosis of GDM (normally from 24 to 36 weeks gestation) from two public maternity hospitals in Kowloon Central Cluster.

During the antenatal visit after the diagnosis of GDM, eligible participants will be approached and invited to join the study. Participants’ socio-demographic, maternal data and planned method of infant feeding will be collected by structured questionnaire at recruitment.

A feasibility and acceptability study of AME on GDM women will be conducted. Planning intervention strategies for intervention group, including conducting survey of the women’s views on antenatal expression practice. A pilot study on antenatal milk expression on GDM women and breastfeeding education will be conducted. Then, development of AME practice include patient information sheet includes the instructions on indication of AME, handling, storage and transportation of expressed milk to hospital, clinical practice guidelines and structured questionnaires.

A qualitative interview will be conducted to explore women’s experience and perceptions on the AME, acceptability and feasibility of the practices compare to standard care. The effect size of AME on GDM women will be examined after the pilot study.

A blocked randomized, parallel-armed controlled trial will be conducted. A computerized random numbers and block sizes of four or six distributed randomly from the Research Randomizer software will be generated.^62^ Single blinded with masked the participants for group allocation and outcome assessor is masked to blocked size and allocation. The investigators deliver the intervention and analysing the data is not blinded. The allocation ratio is 1:1. Allocation concealment use sequentially numbered opaque sealed envelope.

Phase II

This will employ sequential mixed method study design which integrating with quantitative and qualitative study. A simple full scale of randomized controlled trial will be conducted, then qualitative interview for intervention group. A blocked randomized, parallel-armed controlled trial will be conducted. A computerized random numbers and block sizes of four or six distributed randomly from the Research Randomizer software will be generated.^62^ Single blinded with masked the participants for group allocation and outcome assessor is masked to blocked size and allocation. The investigators deliver the intervention and analysing the data is not blinded. The allocation ratio is 1:1. Allocation concealment use sequentially numbered opaque sealed envelope. The cluster trial design and implementation of the study will be guided by CONSORT 2010 guidelines. Reporting and flow diagram of this study will be adopted in accordance with CONSORT statements.^63^ The research protocol will be registered with ClinicalTrials.gov.

**Data sources**

Data collection will consist of data collected from participants’ medical record and directly from participants while visiting obstetric outpatient setting or breastfeeding clinic after their GDM diagnosis and by telephone in the postpartum period. Data collection instruments and structured questionnaire will be developed and will be modified by the PI in the first phase of the study. There will be five data collection tools used in the study as outlined below:

- Maternal and Family Demographic Data (Sociodemographic): Maternal and demographic data such as education, income, employment, family history of diabetes and pre-pregnancy BMI, previous breastfeeding experience will be collected. Mothers will be asked to complete the questionnaire while attending antenatal clinic (IGT/GDM) after eligible participants agreed to join the study and the written consent is signed. This form has been developed into Chinese.
- Maternal and Infant Birth Data: Maternal and infant data will include delivery, infant and birth outcome, and antenatal GDM, infant hypoglycaemia and postnatal fasting blood sugar at 6 weeks of postpartum. The researcher will extract this data from the participant’s medical record.
- Infant Feeding Data: Infant feeding pattern is categorised into three groups including exclusive breastfeeding/ breastmilk feeding (feeding with breastmilk only), mixed feeding or artificial feeding will be collected prevalence before hospital discharge. The data can be retrieved by medical record. The infant feeding pattern will be assessed at 4 and 8 weeks postpartum by telephone follow-up regarding to group allocation by research assistant. The research assistant was blinded for the allocation of the intervention and control group. If women stop breastfeeding, the breastfeeding duration will be measured as the numbers of days of breastfeeding.
- Blood Glucose and Baby admission Data: GDM women will be blood tested for fasting blood sugar at 6 weeks postpartum. The result of the test will be retrieved from participant’s medical record.

**Study Outcomes**

**Phase I**

**Quantitative part**

Primary study outcome:

1) Outcomes will inform if a future definitive RCT of a Gestational Diabetes and Antenatal Human Milk Expression (GAME) Programme in Hong Kong Chinese Women is feasible. Feasibility study outcomes will inform if it is feasible to recruit women to the study; if the women are prepared to be randomized; if women will complete each stage of follow-up at 4 weeks and 8 weeks postpartum; if there are possible sources of contamination which would need to be addressed; women’s views of the intervention offered.
2) Explore and understand maternal experience, perception, and attitude for AME practices.

Secondary study outcome:

Investigating the difference between intervention and control group will be performed for:

1) Examine Breastfeeding Self -Regulation Questionnaire (BSRQ)^54^

The Chinese version of the BSRQ was psychometric tested valid and reliable tool to measure maternal self-determination towards breastfeeding. The Cronbach's alpha of the BSRQ was 0.86. For every one-point increase in the BSRQ score, participants had 15% higher odds of any breastfeeding (OR=1.15, 95%CI 1.07–1.23) and 9% higher odds of exclusive breastfeeding (OR=1.09, 95%CI 1.02–1.17) at 6 weeks postpartum.

2) Measure Breastfeeding Self-Efficacy Scale–Short Form (BSES-SF) when subjects recruit by the questionnaire, at 4 week and at 8 weeks postpartum when woman is breastfeeding using semi-structured questionnaire by phone interview or via Web-based questionnaires. BSES-SF is used to measure maternal breastfeed confidence during postpartum.^60,73^ This instrument consists of 14 items, ranging from 1 = 'Not at all confident' to 5= 'Very confident.' The summary score from 14 to 70 is created by adding 14 items. The scale is psychometrically tested, demonstrates a consistent high reliability and validity. Low breastfeeding self- efficacy is significant predictor of early attrition of breastfeeding.^56,58-59,74-75^
3) Measure Edinburgh Perinatal/Postnatal Depression Scale (EPDS) during antepartum and postpartum.^76-77^ It consist of 10 items include anxiety and depressive and symptoms; cut-off point is 9/10. It has a sensitivity of 0.86 and specificity of 0.78 with a positive predictive value of 73%. It was translated in Hong Kong Chinese. It psychometrically tested, high reliability and validity for perinatal and at 6 weeks postpartum.^77-78^ It will be collected at baseline, on discharge and at 8 weeks postpartum between the two groups. Successful breastfeeding is predictive of lower maternal depressive symptomology.
4) Determine the prevalence of Impaired Glucose Tolerance (IGT). Prevalence of IGT among participants according to breastfeeding status (any breastfeeding or exclusive formula feeding) will be collected from medical record for the fasting blood sugar testing at 6 weeks postpartum.
5) Explore and understand maternal experience, perception, and attitude for AME practices.

**Phase II**

Quantitative part

Primary study outcome:

The exclusive breastfeeding prevalence on discharge, at 4 weeks and 8 weeks postpartum in the intervention and control group

Secondary study outcome:

The difference between intervention and control group will be performed for:

1)	Breastfeeding Self -Regulation Questionnaire (BSRQ)

2)	Breastfeeding self-efficacy scale- Short Form (BSES-SF)

3)	Edinburgh Perinatal/ Postnatal Depression Scale (EPDS)

4)	Maternal experience, perception, and attitude for AME practices and GAME programme.

5)	The prevalence of Impaired Glucose Tolerance (IGT) at 6 weeks postpartum.

Potential Confounders: Data will be collected to measure the following covariates that have been documented to be potentially confounding variables: maternal age, parity, pre-pregnancy BMI, previous GDM, education, family income, employment status, smoking status, previous breastfeeding experience, infant birth weight, baby admission of neonatal unit and mode of delivery and family support and antenatal breastfeeding education.

**Qualitative interview**

Procedure and Data Generation

Semi-structured interview will be conducted. Sample will be recruited by purposive sampling. A pilot study is conducted to familiar with the procedures, explore the skills in conducting an interview. It assesses the questions are comprehended by the participants. Modification of the technique or approach may be required. Interviews are conducted in the breastfeeding clinic or meeting rooms in hospital at around 6 weeks postpartum.

Research Question(s) in the qualitative study will be developed in four core topics include:

1.What is your belief or opinion on the gestational diabetes on breastfeeding?

2.What is your experience on antenatal milk expression (AME)?

3.How are your perception and feelings on AME?

4.What are the viewpoints on AME on breastfeeding and self-efficacy of breastfeeding?

Interview guide will be developed before the interview. Semi-structured interviews will be conducted by the researcher in scheduled breastfeeding clinic or zoom meeting. Interview questions are designed to explore women’s thoughts and experiences about gestational diabetes on breastfeeding, antenatal expression, and their positive and negative feelings on hand expression skills, breastmilk collection and storage. Women are also asked to reflect on the perceptions on the perceived benefits, concerns, practice instructions and use of breastmilk after baby birth. Participants will be willing to disclose themselves and obtain more information after the rapport is established between participants and interviewer in the interview.

Women with varied scores of Breastfeeding self-efficacy scale will be approached via a telephone call to fix the time for interviews to evaluate the experience and perceptions on AME. Women are obtained written consent prior to semi structured interview. Participants will be reminded that they can withdraw from the study if they desire, and anonymity are utilized to protect the privacy. Participants are explained that there are no right or wrong way to share their story. An open and relax atmosphere is created in the interview to allow participants to express without constraint. The interview is a conversation with no leading questions.79 Prompts are given where necessary to encourage women to explore and describe their daily experience on AME and her feelings from her breast and breastfeeding. Questions are restated and are summarized by the researcher to ensure data accuracy. The interview will last approximately 60-90 minutes. An audit trail is utilized in this study using field notes for observations. Memo writing will be taken in the interview to ensure consistency. Follow up interviews will be conducted for ensuring extra meaning from participants.79 Each participant may be interviewed two to three times to enhance interpretation. Interviews are audio-recorded and transcribed verbatim by the researcher. The audio transcription will be read many times and verified for accuracy by researcher, research assistants or peer reviewers. The interview transcript includes body language, voice tone or facial expression.

# 4 Selection and Withdrawal of Subjects

**Study population**

According to the reference of the World Health Organisation 2013, diagnosis of gestational diabetes mellitus and diabetes in pregnancy by 75g Oral Glucose Tolerance Test for risks of pregnancy and risks of diabetes e.g. family history of diabetes. GDM is diagnosed with fasting glucose 5.1-6.9 mmol/L or 8.5-11.0 mmol/L at 2 hours of glucose. Diabetes in pregnancy is diagnosed if fasting glucose >7mmol/L and >11.1mmol/L at 2 hours glucose. Woman is excluded if she diagnoses prior to pregnancy e.g. pre-existing diabetes or diabetes in pregnancy on insulin. Participants who diagnosed with gestational diabetes in pregnancy and plan to delivery in either 2 public hospitals (Kwong Wah Hospital or Queen Elizabeth Hospital) in Hospital Authority Kowloon Central Cluster will be invited to participate at 34-36 week of gestation in the antenatal clinic.

Included eligible participants will be: 1) diagnosed gestational diabetes without on insulin; 2) aged 18 years or above; 3) can read and speak Chinese; 4) Hong Kong resident and stay in Hong Kong after delivery; 5) no serious medical or obstetrical complications; 6) no mental health illness or history; 7) have regular antenatal check-up; 8) singleton pregnancy; 9) primiparous or multiparous women who intention to breastfeed; and 10) pre-pregnancy BMI<25.

Excluded participants will be 1) they do not have Hong Kong Identify Card; 2) she is breastfeeding their elder child at pregnancy; 3) cannot understand the study; 4) give birth <37 weeks of gestation; and 5) a known fetal anomaly or any suspicion of fetal compromise.

Semi-structured interview

Purposive sampling is adopted in the pilot and qualitative study in the second phase. Participants will be recruited by the criterion sampling that meet the criteria. Selecting interviewee is based on their mean difference breastfeeding self-efficacy score will situate breastfeeding self-efficacy as the criterion reference. Five participants will be invited to join the interview. Those participants response the highest and lowest self-efficacy score at baseline and 4 weeks postpartum will be invited to join the interview. Interview guide will be developed for the questions in the interview. It will take approximately 60-90 minutes for the interview. The interview will be conducted in the breastfeeding clinic or zoom meeting in hospital based on participants’ preference.

Participation will be completely voluntary, and participants can be withdrawn from the study at any time.

# 5 Treatment of Subjects

**Intervention**

Study participants will be randomized to standard hospital antenatal care or standard care plus an antenatal expression education*.*

Intervention group will have one face to face contact in lactation clinic and telephone phone follow till delivery. The theory-based intervention “Gestational Diabetes and Antenatal Milk Expression” (GAME) will be formulated based on established protocol used in previous studies^39,49,64^ and experiences of overseas.^51,66-72^ The structured protocols are developed for GDM women and breastfeeding education enhancing breastfeeding self-efficacy. Participants will arrange a face-to-face breastfeeding education in Midwife clinic (Lactation) at 37 weeks of gestation. Behavioural intervention on antenatal milk expression and breastfeeding self-efficacy enhancement including setting their goals, identify their barriers, strengths, and gains in the first and subsequent session of education. The session will approximately last for 45minutes. GDM leaflet and GAME fact sheet are used to facilitate the teaching. The benefits of breastfeeding for women with GDM will be addressed. Maternal metabolic control, calories consumed, and weight loss are addressed for the extra benefits of breastfeeding for GDM women. The possible challenges of GDM on breastfeeding will be support. They will discuss the experience of breastfeeding. The antenatal human milk expression will be introduced.

Besides, video of hand expression of breastmilk is included in the content. Participants will be shown the hand expression video in the clinic. After watching the video, researcher will coach participants on hand expression skills. Participants are instructed to practice antenatal expressing colostrum after 37 weeks of gestation till delivery at home in addition to the conventional care offered by the hospital. The skill is taught by trained midwife or International Board-Certified Lactation Consultation. GDM leaflet with associated website, QR code and linked video leaflets will be given to participants. Video of hand expression of breastmilk, skills on breastfeeding positioning and attachment are shown in the clinic.

Women will be encouraged to express colostrum twice daily for up to 10 minutes until admitted to hospital to give birth. Participants is instructed to engage AME practice at home and record the practice in a written diary in GAME fact sheet. Participants will be asked to record the frequency and duration of the expression session and volume of colostrum. They will receive teaching antenatal expression together with collection kits including syringes and labels. Participants collect the colostrum in syringes. Expressed colostrum will be labelled with women’s name, expression time and hospital medical record number and kept in syringes at home. They are instructed to transport the frozen milk when they go to hospital. They will receive positive reinforcement from researcher when participants can demonstrate correct hand expression skill. Researchers will develop and provide GAME fact sheet on the handling, storage, and instruction on the AME. A log sheet in the fact sheet will be kept ensuring consistency in information delivery. Secondly, self-efficacy and key self-regulatory strategies are adopted by setting a reasonable, proximal goal, planning, self-monitoring in log record on their practice.

They will receive a weekly phone follows up from 38 weeks before delivery and they can contact the researcher by phone in the information sheet if any questions.

First Telephone follow up at 38 weeks and it is last 20min:

Lactation consultants will review and discuss with participants on human milk expression. The benefits of exclusive breastfeeding and the risks of formula supplementation will be reinforced. Social support from family members will be encouraged.

Second or Booster Telephone follow up 39-40 week of gestation and it is last 20min:

Teaching the self-regulatory strategies, tailored problem-solving skills and positive advice are recommended in the booster session. Participants will identify their gains for knowledge checking, facilitate autonomy and consolidate strengths. The barriers of the practicing the antenatal milk expression will be addressed in the contact sessions. Self-reward and reward will provide for participants’ strengths and gains delivering in the programme. The GAME skills and experiences will be revisited and reinforced. They will share and discuss the strategies on problem solving (stimulate control).

Intervention fidelity and adherence

The intervention will be assessed for fidelity of hand expression skills by asking the participants to return demonstration on her breast or using breast models. They can audio record the practice if they encounter any difficulties and attend the breastfeeding clinic for coaching by lactation consultant. The breastfeeding hotline is offered for the participant if they have any questions on handling and storage of the colostrum. Independent reviewers who are trained to teach hand expression skills. Participants will be facilitated to setting their personal goals and explore with them at the beginning of the intervention. Adherence to the intervention will be monitored through their expression diary. Adherence to the practice can be assessed using a structured questionnaire on the number and duration of AME before delivery in telephone interview.

Control group

Participants will be received standard care include prenatal breastfeeding education at gestation of 24-36 weeks same as intervention group. They will not discuss about antenatal milk expression, breastfeeding self-efficacy and the effects of breastfeeding on GDM women. At the time of recruitment, the breastfeeding health talk on hand expression, breastfeeding attachment and positioning and skin to skin contact and signs of getting enough of breastmilk will be deliver during the antenatal visit in hospital. They will receive a breastfeeding diary using in study hospital (usual care) and GDM leaflets. Phone interview will be conducted at 4 weeks postpartum and 8 weeks postpartum for the questionnaire.

Participants will be asked to complete a data collection form and answer question while in the clinic and to allow the study research assistant to extract blood test results and maternal and infant data from the obstetrical record.

# 6 Assessment of Efficacy

Assessment of the efficacy of the intervention will be done at 4 and 8 weeks postpartum using the criteria specified in the study outcomes.

# 7 Assessment of Safety

This research involves a patient education and support intervention and we do not anticipate any patient safety issues. The person carrying out the intervention is a trained midwife and certified lactation consultant with experience in AME education and supporting breastfeeding and postpartum women. However, as a precaution, both the delivery of the intervention and participant outcomes will be closely monitored by the lead and site PI and the study investigators.

# 8 Statistics

**Data Validation**

All data will be entered using data collection form. All entered data will be assessed for any data entry error. Any record without value or missing data will be double-checked to ensure data is entered correctly. A variety of crosschecks will be adopted to ensure minimizing data entry error. Once the data has been error-checked, it will be computed into IBM SPSS Statistics version 26 for data analysis.

**Data Analysis in quantitative study**

Each estimate will be accompanied by a 95% confidence interval. A 5% level of significance will be used in all statistical tests. Descriptive statistics is presented as mean (SD) for continuous variables and n (%) for categorical variables. Univariate analysis t-test, chi-square test for baseline comparison of sociodemographic variables will be employed. For dichotomous data, chi‐square tests will be used to assess exclusively breastfeeding rate at 4 weeks and at 8 weeks postpartum between groups.

For continuous data in the secondary outcome, an independent two‐sample t test is used to assess differences between groups. Multivariable binary logistic regression analysis will be used to assess outcome variables and to adjust for effects of potential confounders. The Intention to treat approach will be used in main analysis. Generalized mixed model will be employed to assess the interaction effect between groups.

**Data management plan for qualitative study**

The interviews will be tape-recorded. Interview is transcribed verbatim and imported into NVIvO 12 software. An audit trail is utilized using field notes for observations.

Memo writing will be taken in the interview to ensure consistency. Inductive thematic analysis is used until data saturation. A categorical analysis by item (or groups of items). Data will be verified, coded and decoded for analysis.

**Sample Size Calculation:**

Phase I

Sample: Total of 20 subjects (10 from interventions and 10 from control) will be recruited from two hospitals.

Qualitative interview

Participants is administered the semi-structured questionnaire for demographic data and experience of antenatal expression. Women are recruited for the semi-structured interview in the postnatal ward before hospital discharge or by phone after discharge. Five interviews will be conducted and invited participants from AME group. The sample size depends on the data saturation or informational redundancy. The sample size is influenced by its ability to provide rich data portraying individual gestational diabetes women’s insights on the impact of AME.

Based on current prevalence rates of GDM, we estimate that approximately 14-15% of pregnant women in the study hospitals will have GDM. One study hospital has approximately 330 births per month and the other approximately 300 per month, resulting in approximately 50 patients per month with GDM at one study site. Approximately 10% would be ineligible because of participant selection criteria, leaving approximately 86 eligible GDM patients. Using a conservative participation rate of approximately 60%, we should be able to recruit about 25 patients per month from both study sites. Therefore, we will be able to fully follow-up approximately 20 patients per month.

Phase II:

Quantitative part

G*power version 3.1.9.4 software is used. Sample size is estimated investigating the effect of AME on exclusive breastfeeding prevalence.

It is assumed to detect a minimal clinical significance difference of 20% in exclusive breastfeeding prevalence of the intervention. Two studies (Forster, 2017; Casey, 2019) are referred indicating the assumed difference is valid. According to a RCT study (Forster, 2014,2017), 69% of women exclusive breastfed versus 60% women of exclusive breastfeed in standard group for first 24 hour with adjusted relative risk of 1.15 (95% CI 1.02-1.28).

Another retrospective study (Casey, 2019) found that 84% of women in AME group exclusive breastfed at discharge compared to 58% women did not. From two studies (Forster, 2017; Casey,2019), the effect size ranged from 9-26%. Hence, we assume a difference of 20% to be detected in the proposed study. From the Breastfeeding Survey from Department of Health in 2019, exclusive breastfeeding prevalence was 32.6% at first month. Whereas the exclusive breastfeeding prevalence at one month postpartum was 30-42% in the study cluster from the Breastfeeding Survey Report in Hospital Authority in 2013. A local survey on done in study site in 2015, breastfeeding prevalence with lactation support shown 20-30% change difference at 4 weeks postpartum.81 Hence, postpartum breastfeeding prevalence of 40% of the control group and 60% of intervention group will be used for sample size calculation in this proposed study. Taking significance level of 5%, power of 80%, and potential attrition rate of 20%, estimated sample size of 244 (97x2/0.8) participants, with 122 in each group based on z test is required for the proposed simple RCT study.

# 9 Direct Access to Source Data/Documents

Once data is collected, only the PI will have access to named data and this will be kept locked in the PIs. Access to the study data will be available for study related monitoring, audit and regulatory inspection by appropriate bodies. It will also be available for IRB review as appropriate.

# 10 Quality Control and Quality Assurance

Analysis will be reviewed by the study team to ensure it is appropriate, accurate and correctly interpreted.

# 11 Ethics

The main ethical considerations for this study are to ensure confidentiality to the participants and to ensure that the participants feel free to either participate in the study or to not participate in the study. The following measures will be taken to address these concerns:

- Participation in the study will be completely voluntary and potential participants will be assured that they will not suffer any consequences of lower standards of care for not participating.
- Participants will be also informed that they may withdraw from the study at any time without any repercussions.
- Informed written consent will be obtained from all participants.
- No specific identifiers (such as name, ID number, Hospital number, bed number, telephone number and address) will be included in the data used for analysis or be available to the study team. Other study information will not be specific enough, either singly or in combination, to uniquely identify an individual.
- Audio/Videotape will be recorded during the interviews in the case studies but no participant identifier such as names will be mentioned in the interviews. The record will be kept about three years after completion of the data collection or public release the work of the research. Study ID number will be appeared in the record. The record will be permanently deleted after the completion of the study.

This study will be complied with the Declaration of Helsinki (version: 2000) and ICH-GCP.

# 12 Data Handling and Record Keeping

Anonymised data will be analysed within study period. All data collection forms will contain a study ID number only. The only data which will be able to link the participant with the study data is the consent form. All signed consent forms will be kept in the PI in a locked filing cabinet. All video or audio recording will be stored in PI’s computer with password protected.

# 13 Financing and Insurance

This research did not receive any funding.

# 14 Publication Policy

Papers approved by the study team will be published in appropriate peer reviewed journals.

**16. References**

1.	Cho NH, Shaw JE, Karuranga S, Huang Y, da Rocha Fernandes JD, Ohlrogge AW, et al. IDF Diabetes Atlas: Global estimates of diabetes prevalence for 2017 and projections for 2045. Diabetes Res Clin Pract. 2018;138:271-81.

2.	International Diabetes Federation. Diabetes atlas (9th ed.) Brussels. 2019. p. https://diabetesatlas.org/.

3.	Saeedi P, Petersohn I, Salpea P, Malanda B, Karuranga S, Unwin N, et al. WITHDRAWN: Global and regional diabetes prevalence estimates for 2019 and projections for 2030 and 2045: results from the International Diabetes Federation Diabetes Atlas, 9(th) edition. Diabetes Res Clin Pract. 2019:107843.

4.	Organization WH. Global health estimates 2021 [Available from: www.who.int/data/global health estimates.

5.	Tam WH, Ma RCW, Ozaki R, Li AM, Chan MHM, Yuen LY, et al. In Utero Exposure to Maternal Hyperglycemia Increases Childhood Cardiometabolic Risk in Offspring. Diabetes Care. 2017;40(5):679-86.

6.	The Hong Kong College of Obstetricians and Gynaecologists AFCoHKAoM. Guidelines for the Management of Gestational Diabetes Mellitus updated. HKCOG Guidelines. 2016;Number 7.

7.	Bellamy L, Casas JP, Hingorani AD, Williams D. Type 2 diabetes mellitus after gestational diabetes: a systematic review and meta-analysis. Lancet. 2009;373(9677):1773-9.

8.	Lee KF, Mak MW, Lau KO, Chung H. Risk of development of diabetes mellitus in Chinese women with persistently impaired glucose tolerance after gestational diabetes. Hong Kong Med J. 2011;17(3):195-201.

9.	Fallon A, Dunne F. Breastfeeding practices that support women with diabetes to breastfeed. Diabetes Res Clin Pract. 2015;110(1):10-7.

10.	Hui LL, Kwok MK, Nelson EAS, Lee SL, Leung GM, Schooling CM. The association of breastfeeding with insulin resistance at 17 years: Prospective observations from Hong Kong's "Children of 1997" birth cohort. Matern Child Nutr. 2018;14(1).

11.	Jagiello KP, Azulay Chertok IR. Women's Experiences With Early Breastfeeding After Gestational Diabetes. J Obstet Gynecol Neonatal Nurs. 2015;44(4):500-9.

12.	Much D, Beyerlein A, Rossbauer M, Hummel S, Ziegler AG. Beneficial effects of breastfeeding in women with gestational diabetes mellitus. Mol Metab. 2014;3(3):284-92.

13.	McManus RM, Cunningham I, Watson A, Harker L, Finegood DT. Beta-cell function and visceral fat in lactating women with a history of gestational diabetes. Metabolism: clinical and experimental. 2001;50(6):715-9.

14.	Shub A, Miranda M, Georgiou HM, McCarthy EA, Lappas M. The effect of breastfeeding on postpartum glucose tolerance and lipid profiles in women with gestational diabetes mellitus. Int Breastfeed J. 2019;14:46.

15.	Doughty K, Taylor SN. Barriers and benefits to breastfeeding with gestational diabetes. Semin Perinatol. 2021:151385.

16.	Mannel R, Martens PJ, Walker M. Core Curriculum for Lactation Consultant Practice. 3 ed. Association ILC, editor. United States of America: Kevin Sullivan, Jones & Bartlett Learning; 2013.

17.	Walker M. Breastfeeding management for the clinician : Using the evidence. Third ed. United States of America: Jones and Bartlett Learning; 2014.

18.	Aune D, Norat T, Romundstad P, Vatten LJ. Breastfeeding and the maternal risk of type 2 diabetes: a systematic review and dose-response meta-analysis of cohort studies. Nutr Metab Cardiovasc Dis. 2014;24(2):107-15.

19.	Horta BL, de Lima NP. Breastfeeding and Type 2 Diabetes: Systematic Review and Meta-Analysis. Curr Diab Rep. 2019;19(1):1.

20.	Feng L, Xu Q, Hu Z, Pan H. Lactation and progression to type 2 diabetes in patients with gestational diabetes mellitus: A systematic review and meta-analysis of cohort studies. J Diabetes Investig. 2018;9(6):1360-9.

21.	Gunderson EP, Hurston SR, Ning X, Lo JC, Crites Y, Walton D, et al. Lactation and Progression to Type 2 Diabetes Mellitus After Gestational Diabetes Mellitus: A Prospective Cohort Study. Ann Intern Med. 2015;163(12):889-98.

22.	Gunderson EP, Lewis CE, Lin Y, Sorel M, Gross M, Sidney S, et al. Lactation Duration and Progression to Diabetes in Women Across the Childbearing Years: The 30-Year CARDIA Study. JAMA Intern Med. 2018;178(3):328-37.

23.	Shen Y, Leng J, Li W, Zhang S, Liu H, Shao P, et al. Lactation intensity and duration to postpartum diabetes and prediabetes risk in women with gestational diabetes. Diabetes Metab Res Rev. 2019;35(3):e3115.

24.	Song C, Lyu Y, Li C, Liu P, Li J, Ma RC, et al. Long-term risk of diabetes in women at varying durations after gestational diabetes: a systematic review and meta-analysis with more than 2 million women. Obes Rev. 2018;19(3):421-9.

25.	Ley SH, Chavarro JE, Li M, Bao W, Hinkle SN, Wander PL, et al. Lactation Duration and Long-term Risk for Incident Type 2 Diabetes in Women With a History of Gestational Diabetes Mellitus. Diabetes Care. 2020;43(4):793-8.

26.	Service FH. Breastfeeding Survey 2019 Department of Health; 2019 [Available from: www.fhs.gov.hk/english/archive/files/reports/BF_survey_2019.pdf.

27.	Victora CG, Bahl R, Barros AJ, França GV, Horton S, Krasevec J, et al. Breastfeeding in the 21st century: epidemiology, mechanisms, and lifelong effect. Lancet. 2016;387(10017):475-90.

28.	Organization {Organization, 2014 #157} WH. Global nutrition targets 2025: breastfeeding policy brief (WHO/NMH/NHD/14.7). Geneva2014 [WHO/UNICEF]. Available from: https://apps.who.int/iris/bitstream/handle/10665/149022/WHO_NMH_NHD_14.7_eng.pdf?ua=1.

29.	Matias SL, Dewey KG, Quesenberry CP, Jr., Gunderson EP. Maternal prepregnancy obesity and insulin treatment during pregnancy are independently associated with delayed lactogenesis in women with recent gestational diabetes mellitus. Am J Clin Nutr. 2014;99(1):115-21.

30.	Doughty KN, Ronnenberg AG, Reeves KW, Qian J, Sibeko L. Barriers to Exclusive Breastfeeding Among Women With Gestational Diabetes Mellitus in the United States. J Obstet Gynecol Neonatal Nurs. 2018;47(3):301-15.

31.	Finkelstein SA, Keely E, Feig DS, Tu X, Yasseen AS, 3rd, Walker M. Breastfeeding in women with diabetes: lower rates despite greater rewards. A population-based study. Diabet Med. 2013;30(9):1094-101.

32.	Oza-Frank R, Moreland JJ, McNamara K, Geraghty SR, Keim SA. Early Lactation and Infant Feeding Practices Differ by Maternal Gestational Diabetes History. J Hum Lact. 2016;32(4):658-65.

33.	De Bortoli J, Amir LH. Is onset of lactation delayed in women with diabetes in pregnancy? A systematic review. Diabet Med. 2016;33(1):17-24.

34.	Nguyen PTH, Binns CW, Nguyen CL, Ha AVV, Chu TK, Duong DV, et al. Gestational Diabetes Mellitus Reduces Breastfeeding Duration: A Prospective Cohort Study. Breastfeed Med. 2019;14(1):39-45.

35.	East CE, Dolan WJ, Forster DA. Antenatal breast milk expression by women with diabetes for improving infant outcomes. Cochrane Database Syst Rev. 2014(7):CD010408.

36.	Chertok IR, Sherby E. Breastfeeding Self-efficacy of Women With and Without Gestational Diabetes. MCN Am J Matern Child Nurs. 2016;41(3):173-8.

37.	Oza-Frank R, Gunderson EP. In-Hospital Breastfeeding Experiences Among Women with Gestational Diabetes. Breastfeed Med. 2017;12:261-8.

38.	Loewenberg Weisband Y, Rausch J, Kachoria R, Gunderson EP, Oza-Frank R. Hospital Supplementation Differentially Impacts the Association Between Breastfeeding Intention and Duration Among Women With and Without Gestational Diabetes Mellitus History. Breastfeed Med. 2017;12(6):338-44.

39.	Forster DA, McEgan K, Ford R, Moorhead A, Opie G, Walker S, et al. Diabetes and antenatal milk expressing: a pilot project to inform the development of a randomised controlled trial. Midwifery. 2011;27(2):209-14.

40.	Cox SG. Expressing and storing colostrum antenatally for use in the newborn period. Breastfeeding Review. 2006;14(3):6.

41.	Foudil-Bey I, Murphy MS, Keely EJ, El-Chaâr D. Maternal and newborn outcomes of antenatal breastmilk expression: a scoping review protocol. BMJ Open. 2020;10(5):e033101.

42.	Casey JRR, Banks J, Braniff K, Buettner P, Heal C. The effects of expressing antenatal colostrum in women with diabetes in pregnancy: A retrospective cohort study. Aust N Z J Obstet Gynaecol. 2019.

43.	Singh G, Chouhan R, Sidhu K. Effect of Antenatal Expression of Breast Milk at Term in Reducing Breast Feeding Failures. Medical Journal Armed Forces India. 2009;65(2):131-3.

44.	Brisbane JM, Giglia RC. Experiences of expressing and storing colostrum antenatally: A qualitative study of mothers in regional Western Australia. J Child Health Care. 2013;19(2):206-15.

45.	Chapman T, Pincombe J, Harris M, Fereday J. Antenatal breast expression: exploration and extent of teaching practices amongst International Board Certified Lactation Consultant midwives across Australia. Women Birth. 2013;26(1):41-8.

46.	Chapman T, Pincombe J, Harris M. Antenatal breast expression: a critical review of the literature. Midwifery. 2013;29(3):203-10.

47.	Forster DA. Is not testing a major change in clinical practice an ethical dilemma? Midwifery. 2011;27:2.

48.	Soltani H, Scott AMS. Antenatal breast expression in women with diabetes outcomes from a retrospective cohort study. International Breastfeeding Journal. 2012;7(18).

49.	Forster DA, Moorhead AM, Jacobs SE, Davis PG, Walker SP, McEgan KM, et al. Advising women with diabetes in pregnancy to express breastmilk in late pregnancy (Diabetes and Antenatal Milk Expressing [DAME]): a multicentre, unblinded, randomised controlled trial. The Lancet. 2017;389(10085):2204-13.

50.	Lamba S, Chopra S, Negi M. Effect of Antenatal Breast Milk Expression at Term Pregnancy to Improve Post Natal Lactational Performance. J Obstet Gynaecol India. 2016;66(1):30-4.

51.	Demirci JR, Glasser M, Fichner J, Caplan E, Himes KP. "It gave me so much confidence": First-time U.S. mothers' experiences with antenatal milk expression. Matern Child Nutr. 2019:e12824.

52.	Fair FJ, Watson H, Gardner R, Soltani H. Women's perspectives on antenatal breast expression: a cross-sectional survey. Reprod Health. 2018;15(1):58.

53.	Casey JRR, Mogg EL, Banks J, Braniff K, Heal C. Perspectives and experiences of collecting antenatal colostrum in women who have had diabetes during pregnancy: a North Queensland semistructured interview study. BMJ Open. 2019;9(1):e021513.

54.	Lau CY, Fong DY, Choi AY, Ng JW, Sing C, Tarrant M. Development and measurement properties of the Chinese breastfeeding self-regulation questionnaire. Midwifery. 2017;44:24-34.

55.	Loke AY, Chan LK. Maternal breastfeeding self-efficacy and the breastfeeding behaviors of newborns in the practice of exclusive breastfeeding. J Obstet Gynecol Neonatal Nurs. 2013;42(6):672-84.

56.	Lau CYK, Lok KYW, Tarrant M. Breastfeeding Duration and the Theory of Planned Behavior and Breastfeeding Self-Efficacy Framework: A Systematic Review of Observational Studies. Matern Child Health J. 2018;22(3):327-42.

57.	Brockway M, Benzies K, Hayden KA. Interventions to Improve Breastfeeding Self-Efficacy and Resultant Breastfeeding Rates: A Systematic Review and Meta-Analysis. J Hum Lact. 2017;33(3):486-99.

58.	Chan MY, Ip WY, Choi KC. The effect of a self-efficacy-based educational programme on maternal breast feeding self-efficacy, breast feeding duration and exclusive breast feeding rates: A longitudinal study. Midwifery. 2016;36:92-8.

59.	Dennis CL. Theoretical underpinnings of breastfeeding confidence: a self-efficacy framework. J Hum Lact. 1999;15(3):195-201.

60.	Dennis CL. The breastfeeding self-efficacy scale: psychometric assessment of the short form. J Obstet Gynecol Neonatal Nurs. 2003;32(6):734-44.

61.	You H, Lei A, Xiang J, Wang Y, Luo B, Hu J. Effects of breastfeeding education based on the self-efficacy theory on women with gestational diabetes mellitus: A CONSORT-compliant randomized controlled trial. Medicine (Baltimore). 2020;99(16):e19643.

62.	Randomizer R. Research Randomizer 2021 [Research Randomizer]. Available from: https://www.randomizer.org/links/.

63.	Moher D, Hopewell S, Schulz KF, Montori V, Gøtzsche PC, Devereaux PJ, et al. CONSORT 2010 explanation and elaboration: updated guidelines for reporting parallel group randomised trials. Int J Surg. 2012;10(1):28-55.

64.	Forster DA, Jacobs S, Amir LH, Davis P, Walker SP, McEgan K, et al. Safety and efficacy of antenatal milk expressing for women with diabetes in pregnancy: protocol for a randomised controlled trial. BMJ Open. 2014;4(10):e006571.

65.	Creswell JW. Qualitative inquiry & research design: Choosing among five approaches. London: Sage Publications; 2007.

66.	O'Sullivan TA, Cooke J, McCafferty C, Giglia R. Online Video Instruction on Hand Expression of Colostrum in Pregnancy is an Effective Educational Tool. Nutrients. 2019;11(4).

67.	District WSLH. Fact Sheet Hand expression of colostrum during pregnancy. In: Hospital WsNHW, editor. 2017.

68.	Giddins H. Antenatal Expression of Colostrum NHS for parents. 2017.

69.	Service BRH. Expressing-Colostrum-antenatally. 2015.

70.	Trust MEHSN. Antenatal Expression of Colostrum Information for Mothers-to-be. 2016.

71.	Trust UHSNF. Harvesting colostrum for your baby. 2017.

72.	Wrightington WaL. Colostrum harvesting/expressing your milk in the antenatal period. NHS Foundation Trust. 4 ed2019. p. 1-6.

73.	Ip WY, Yeung LS, Choi KC, Chair SY, Dennis CL. Translation and validation of the Hong Kong Chinese version of the breastfeeding self-efficacy scale-short form. Res Nurs Health. 2012;35(5):450-9.

74.	Bai YK, Lee S, Overgaard K. Critical Review of Theory Use in Breastfeeding Interventions. J Hum Lact. 2019;35(3):478-500.

75.	Tuthill EL, McGrath JM, Graber M, Cusson RM, Young SL. Breastfeeding Self-efficacy: A Critical Review of Available Instruments. J Hum Lact. 2016;32(1):35-45.

76.	Cox JL, Holden JM, Sagovsky R. Detection of postnatal depression. Development of the 10-item Edinburgh Postnatal Depression Scale. Br J Psychiatry. 1987;150:782-6.

77.	Lee DT, Yip SK, Chiu HF, Leung TY, Chan KP, Chau IO, et al. Detecting postnatal depression in Chinese women. Validation of the Chinese version of the Edinburgh Postnatal Depression Scale. Br J Psychiatry. 1998;172:433-7.

78.	Lee DT, Chan SS, Sahota DS, Yip AS, Tsui M, Chung TK. A prevalence study of antenatal depression among Chinese women. J Affect Disord. 2004;82(1):93-9.

79.	Munhall PL. Interpretive phenomenology. Routledge: Routledge international handbook of qualitative nursing research; 2013.

80.	Braun V, Clarke V. Using thematic analysis in psychology. Qualitative Research in Psychology. 2006;3(2):77-101.

81. Leung, Y. N., Sing C., Yeung, A., Leung, S.., Chow, Y.K, Leung, W.C. (2015) Effectiveness of Lactation Consultant Support on Exclusive Breastfeeding Prevalence After Discharge. Oral presentation in The ICM Asia Pacific Regional Conference and Hospital Authority Convention in 2015.

**17. Supplements Timeline**

|  | Year/months | | | | | | | | | | |
| --- | --- | --- | --- | --- | --- | --- | --- | --- | --- | --- | --- |
| Task | 2021 | 2022 | | | | 2023 | | | | 2024 | |
|  | Oct-Dec | Jan-Mar | Apr-Jun | July-Sep | Oct-Dec | Jan-Mar | Apr-Jun | July-Sep | Oct-Dec | Jan-Mar | Apr-Jun |
| Draft the logistics, guidelines, workflow of intervention |  |  |  |  |  |  |  |  |  |  |  |
| Development of research protocol and apply the REC |  |  |  |  |  |  |  |  |  |  |  |
| Dissemination the intervention |  |  |  |  |  |  |  |  |  |  |  |
| Implement the intervention |  |  |  |  |  |  |  |  |  |  |  |
| Pilot study on questionnaire, interview and pilot RCT |  |  |  |  |  |  |  |  |  |  |  |
| Qualitative interview |  |  |  |  |  |  |  |  |  |  |  |
| Qualitative transcription of the theme, data analysis and interpretation |  |  |  |  |  |  |  |  |  |  |  |
| Writing the report and present the findings to REC |  |  |  |  |  |  |  |  |  |  |  |
| (Phase 2) Full scale of RCT, recruitment and randomisation |  |  |  |  |  |  |  |  |  |  |  |
| Quantitative (RCT) data collection until sample size is met |  |  |  |  |  |  |  |  |  |  |  |
| Qualitative interview |  |  |  |  |  |  |  |  |  |  |  |
| Qualitative transcription of the theme, data analysis and interpretation |  |  |  |  |  |  |  |  |  |  |  |
| Consultation/peer review and evaluation |  |  |  |  |  |  |  |  |  |  |  |
| Writing the thesis report and present the findings |  |  |  |  |  |  |  |  |  |  |  |

**18. Appendix**

1. Participant Information Sheet – Chinese version, version 2, dated Dec 2, 2021
2. Participant Informed Consent– Chinese version dated Dec 2, 2021
3. Baselines Questionnaires-Chinese version, version 2, dated Dec 2, 2021
4. Chinese Questionnaires 4 weeks version 2, dated Dec 2, 2021
5. Chinese Questionnaires 8 weeks version 2, dated Dec 2, 2021
6. Data collection form version 2, dated Dec 2, 2021
7. Telephone one month data
8. Telephone two-month data
9. Interview guide
10. GAME leaflet
11. AME Factsheet
12. Infant feeding Diary
