# Supplementary material for: The Gestational Diabetes Antenatal Milk Expression (GAME) trial: effects on breastfeeding outcomes and maternal mental health—A randomised controlled trial
Source: eClinicalMedicine. 2026 Aug 5;98:104130. doi: 10.1016/j.eclinm.2026.104130 (PMC13463982; doi:10.1016/j.eclinm.2026.104130)
Supplement: Supplementary file 2 [file mmc2.docx]

Supplementary material:

**Contents**

Statistical Analysis Plan

Table.

Table S1. Comparison of baseline characteristics by missing data status

Table S2. Sensitivity analyses for exclusive breastfeeding (GLMM)

Table S3. Exploratory subgroup analysis for exclusive breastfeeding (GLMM)

Table S4. Proportion of women scoring $\boldsymbol{\geq}$ 10 on the EPDS at each time point

Figure

Figure S1. Normality of continuous outcomes using Q-Q plots and Shapiro-Wilk test at each time point.

**STATISTICAL ANALYSIS PLAN**

**Gestational Diabetes and Antenatal Milk Expression (GAME) Trial**

Effects of a structured antenatal milk expression programme on breastfeeding outcomes and maternal mental health in women with gestational diabetes: A Randomised Controlled Trial

| Version | 1.0 (Final) |
| --- | --- |
| Date Finalised | November 2021 (prior to database lock) |
| Trial Registration | ClinicalTrials.gov: NCT05851651 |
| Principal Investigator | Dr Kris Yuet Wan Lok, School of Nursing, The University of Hong Kong |
| Statisticians | Pui Hing Chau, PhD  Ying Dai, PhD |
| Ethics Approval | UW21-364 (HKU/HK Western Cluster); KC/KE-21-0119/ER-1 (KCC/KE REC) |
| Funding | KCC Research Committee (KCC/RG/G/2223-A02); HKU Seed Funding (2202100786) |

This Statistical Analysis Plan (SAP) was finalised prior to completion of data collection and database lock. It pre-specifies all analyses to be conducted, including the hierarchy of primary and secondary models. No modifications have been made to this document after unblinding or analysis.

# **1. Background and Objectives**

**1.1 Background**

Gestational diabetes mellitus (GDM) is a common pregnancy complication associated with increased risk of delayed lactogenesis, suboptimal breastfeeding outcomes, and elevated postpartum depressive symptoms. Antenatal milk expression (AME) has been proposed as an intervention to address these challenges, but robust evidence from randomised controlled trials is lacking, particularly in Asian populations and specifically for women with GDM.

**1.2 Objectives**

The primary objectives of the GAME trial are to evaluate whether a structured AME programme, compared with standard care, improves:

• Co-primary outcome 1: Any breastfeeding rates across the postpartum period

• Co-primary outcome 2: Exclusive breastfeeding rates across the postpartum period

Secondary objectives include evaluating effects on maternal depressive symptoms (EPDS), breastfeeding self-efficacy (BSES-SF), and breastfeeding self-regulation (BSRQ), and confirming the safety of the intervention with respect to neonatal and maternal outcomes.

# **2. Trial Design**

The GAME trial is a two-centre, parallel-group, assessor-blinded, randomised controlled trial conducted at two public antenatal clinics in Hong Kong. Participants were randomly assigned 1:1 to the GAME intervention (structured AME programme plus standard care) or to the control group (standard care only). Stratification was by study site, using computer-generated permuted block randomisation with sequentially numbered, sealed opaque envelopes.

The planned total sample size was 246 participants (123 per arm), calculated a priori to detect an 18 percentage point increase in exclusive breastfeeding rates (from 20% to 38%; Cohen's h = 0.40) with ≥80% power at a two-sided 5% significance level. The trial ran from 3 January 2022 to 27 November 2023.

# **3. Outcomes**

**3.1 Co-Primary Outcomes**

Both of the following outcomes are designated as co-primary outcomes, with equal standing in the hierarchy of confirmatory analyses:

**(i)** Any breastfeeding: defined as providing any breastmilk (including expressed milk) to the infant, irrespective of supplementation, at each of the three postpartum assessment time points (hospital discharge, four weeks, and eight weeks postpartum). Operationalised as a binary variable (yes/no). Missing data will be handled using a worst-case imputation approach: missing values are assumed to represent cessation of breastfeeding (i.e., coded as 0/No).

**(ii)** Exclusive breastfeeding: defined according to World Health Organisation (WHO) criteria as the infant receiving only breastmilk (including expressed milk or milk from a wet nurse) and no other liquids or solids, with the exception of oral rehydration solution, drops, or syrups (vitamins, minerals, medicines), at each of the three postpartum time points. Operationalised as a binary variable (yes/no). The same worst-case missing data assumption applies.

All co-primary analyses are confirmatory. A significance threshold of p < 0.05 (two-sided) will be used to declare statistical significance for primary outcomes, unless otherwise pre-specified by regulatory guidance.

**3.2 Secondary Outcomes**

The following outcomes are secondary, and findings will be interpreted as exploratory/hypothesis-generating rather than confirmatory:

• Maternal depressive symptoms: total score on the Edinburgh Postnatal Depression Scale (EPDS), assessed at baseline, four weeks, and eight weeks postpartum

• Breastfeeding self-efficacy: total score on the Breastfeeding Self-Efficacy Scale – Short Form (BSES-SF, 14 items; range 14–70), assessed at baseline, four weeks, and eight weeks postpartum

• Breastfeeding self-regulation: total score on the Breastfeeding Self-Regulation Questionnaire (BSRQ, 22 items), assessed at baseline, four weeks, and eight weeks postpartum

**3.3 Safety Outcomes**

Neonatal and maternal safety will be assessed through the following endpoints: neonatal hypoglycaemia (blood glucose < 2.6 mmol/L in the first 24 hours), neonatal intensive care unit (NICU)/neonatal unit (NNU) admission, length of NNU stay, baby birth weight, mode of delivery, gestational age at delivery, incidence of uterine contractions or preterm labour following AME initiation, and 6-week postpartum oral glucose tolerance test (OGTT) results.

# **4. Statistical Principles**

**4.1 Analysis Population**

All primary and secondary analyses will be conducted on the intention-to-treat (ITT) population, defined as all randomised participants regardless of adherence to the allocated intervention or completeness of follow-up. Safety outcomes will also be summarised in the ITT population.

**4.2 Significance Level**

For co-primary outcomes, a Bonferroni-corrected two-sided significance threshold of α = 0.025 will be applied. For secondary outcomes, a two-sided α = 0.05 will be used; however, given the exploratory nature of these analyses, findings should be interpreted in the context of multiplicity. No formal correction for multiple comparisons is applied to secondary outcomes.

**4.3 Missing Data**

For the co-primary binary breastfeeding outcomes, missing data will be treated using a worst-case imputation approach: participants with missing outcome data are assumed to have stopped breastfeeding (coded as 0). This approach is conservative and protects against overestimation of breastfeeding rates.

For continuous secondary outcomes (EPDS, BSES-SF, BSRQ), missing data will be handled under the assumption of missing at random (MAR) using the mixed-effects model, which utilises all available data at observed time points without requiring complete case analysis.

**4.4 Baseline Characteristics**

Baseline sociodemographic and clinical characteristics will be described separately for the intervention and control groups using means and standard deviations for continuous variables and frequencies and percentages for categorical variables.

# **5. Primary Outcome Analyses**

The primary analysis for each co-primary outcome will use Generalized Linear Mixed Models (GLMM) with a binomial distribution and logit link function. This approach accounts for the repeated-measures design (three correlated observations per participant) and the binary nature of the outcome, while providing an overall estimate of the treatment effect across the postpartum period.

**5.1 Model Structures**

Two model structures are planed for each co-primary outcome. The hierarchy is as follows:

**Model Structure A (Primary Analysis): Main-effects model.** This model includes fixed effects for treatment group (GAME vs. control), time (discharge, 4 weeks, 8 weeks), and pre-specified baseline covariates, together with a random intercept for each participant to account for within-subject correlation. The GAME treatment effect (aOR with 95% CI and p-value) is estimated as the average treatment effect across all three time points. This model corresponds to Models 1 and 3 in Table 4 of the manuscript (for any breastfeeding and exclusive breastfeeding, respectively), and represents the pre-specified primary analysis for each co-primary outcome.

**Model Structure B (Secondary/Exploratory Analysis): Interaction model.** This model extends Model Structure A by including a group-by-time interaction term. This allows examination of whether the treatment effect varies across the three time points (i.e., whether the GAME intervention has a differential effect at discharge, four weeks, and eight weeks postpartum). This model corresponds to Models 2 and 4 in Table 4 of the manuscript. Because this model cannot yield a single summary treatment effect estimate, it is designated as exploratory.

**5.2 Covariates**

All multivariable models (GLMM and LMM) will adjust for the following baseline covariates, which were selected a priori based on clinical knowledge and their established association with breastfeeding outcomes:

• Study site (hospital)

• Maternal education level

• Return to work postpartum (planned)

• Intention to breastfeed (duration)

• Parity

• Breastfeeding support from family

**5.3 Time-Point–Specific Supplementary Analysis**

In addition to the GLMM, multivariable logistic regression will be conducted at each individual time point (hospital discharge, four weeks postpartum, and eight weeks postpartum) to provide adjusted odds ratios for the treatment effect at each assessment. These time-point–specific analyses are supplementary to, not substitutes for, the primary GLMM analysis.

**5.4 Summary of Model Hierarchy**

| **Model** | **Outcome** | **Structure** | **Designation** | **Table reference** |
| --- | --- | --- | --- | --- |
| 1 | Any breastfeeding | Main effects (no interaction) | Primary analysis | Table 4, Model 1 |
| 2 | Any breastfeeding | Group × Time interaction | Secondary/exploratory | Table 4, Model 2 |
| 3 | Exclusive breastfeeding | Main effects (no interaction) | Primary analysis | Table 4, Model 3 |
| 4 | Exclusive breastfeeding | Group × Time interaction | Secondary/exploratory | Table 4, Model 4 |

All four models are adjusted for the pre-specified covariates listed in Section 5.2. Results will be presented as adjusted odds ratios (aOR) with 95% confidence intervals and two-sided p-values.

# **6. Secondary Outcome Analyses**

**6.1 Continuous Secondary Outcomes: EPDS, BSES-SF, BSRQ**

For the three continuous secondary outcomes (EPDS, BSES-SF, and BSRQ), assessed at baseline, four weeks, and eight weeks postpartum, the following analytical approach will be applied:

***Step 1: Descriptive analysis***

Means, standard deviations (SD), and standard errors (SE) will be reported separately for each group at each time point.

***Step 2: Independent t-tests***

As a preliminary analysis, independent samples t-tests will be conducted at each time point (baseline, four weeks, eight weeks) to characterise unadjusted group differences. These serve a descriptive purpose and are not the primary inferential approach.

***Step 3: Linear Mixed Models (LMM) — primary inferential approach***

The primary longitudinal analysis for each continuous secondary outcome will use a Linear Mixed Model (LMM) with a random intercept for each participant. Fixed effects will include treatment group, time, the group-by-time interaction, and the same pre-specified covariates as the primary GLMM. Marginal mean estimates, mean differences (with 95% CI and p-values), and interaction estimates (β coefficients) will be reported.

The LMM for each outcome will include the corresponding baseline measurement as a covariate, to improve precision and control for potential baseline imbalance.

**6.2 Safety Outcomes**

Safety outcomes will be presented descriptively using frequencies and percentages (for categorical outcomes) or means and SDs (for continuous outcomes). Between-group differences will be assessed using chi-squared tests (or Fisher's exact tests where cell counts < 5) for categorical outcomes and independent samples t-tests for continuous outcomes. These analyses are descriptive and no adjustments for multiplicity will be applied.

# **7. Subgroup and Sensitivity Analyses**

**7.1 Pre-specified Subgroup Analyses**

No formal subgroup analyses are pre-specified as primary analyses in this SAP. Any subgroup analyses conducted post-hoc will be clearly labelled as exploratory and interpreted accordingly.

**7.2 Sensitivity Analysis: Complete Case**

A complete-case sensitivity analysis will be conducted for both co-primary outcomes, restricted to participants who completed all three time points without missing outcome data, to examine the robustness of findings under a missing-completely-at-random assumption.

# **8. Handling of Baseline Characteristics Table**

In accordance with CONSORT 2010 reporting guidelines (Moher et al., 2010) and the recommendation of Altman (1985), significance tests will not be applied to baseline characteristics. The purpose of the baseline table is to describe the study population and evaluate whether randomisation has produced broadly comparable groups; it is not to test the null hypothesis of no difference (which is known to be true by design). Any apparent numerical imbalances should be attributed to chance. Where notable imbalances are observed, these will be acknowledged in the text and considered as potential confounders to be addressed through the adjusted analyses.

# **9. Reporting of Results**

Estimated effects will be presented as odds ratios (OR) or mean differences with 95% confidence intervals (CI) and two-sided p-values. Exact p-values will be reported throughout, rather than inequality statements (e.g., p = 0.023 rather than p < 0.05), except where p < 0.001, in which case the convention p < 0.001 will be used.

# **10. Software**

Statistical analyses will be conducted using R (version 4.0.0; R Core Team, Vienna, Austria) and SPSS version 26.0 (IBM Corp, Armonk, NY, USA). GLMMs and LMMs will be fitted using the lme4 and lmerTest packages in R. Estimated marginal means and pairwise comparisons will be obtained using the emmeans package.

**Table S1. Comparison of Baseline Characteristics by Missing Data Status**

|  | **Complete outcome data (n = 210)** | **Any missing outcome data (n = 36)** | **P value** |
| --- | --- | --- | --- |
| Maternal age, y, mean (SD) | 34.6 (4.37) | 34.5 (4.65) | 0.90 |
| BMI, kg/m², mean (SD) | 22.9 (3.28) | 22.0 (3.06) | 0.20 |
| Intervention group, No. (%) | 108 (51.4) | 15 (41.7) | 0.30 |
| Multiparous, No. (%) | 113 (53.8) | 19 (52.8) | 1.00 |
| Diploma/degree or above, No. (%) | 121 (57.6) | 12 (33.3) | 0.10 |

**Note:** P values from independent t-tests (continuous) or chi-square tests (categorical). None reached statistical significance, supporting the assumption that missing data were missing at random.

**Table S2. Sensitivity Analyses for Exclusive Breastfeeding (GLMM)**

| **Analysis** | **N** | **aOR (95% CI)** | **P value** | **Interpretation** |
| --- | --- | --- | --- | --- |
| **ITT with worst-case imputation (primary)** | 246 | 2.43 (1.17–5.04) | 0.02 | Most conservative; missing = cessation |
| Complete-case analysis | 210 | 3.06 (1.25–7.51) | 0.014 | Excludes participants with any missing BF data |

**Note:** aOR = adjusted odds ratio; BF = breastfeeding; ITT = intention-to-treat. All models used GLMM with logit link, random intercept for participant, adjusted for study site, maternal education, parity, return to work postpartum, intention to breastfeed, and breastfeeding support. No significant differences in baseline characteristics were found between participants with complete (n = 210) and missing (n = 36) outcome data (age p = 0.9; BMI p = 0.2; group allocation p = 0.3; parity p = 1.0; education p = 0.1), supporting the missing at random assumption.

**Table S3. Exploratory subgroup analysis for exclusive breastfeeding (GLMM)**

| **Subgroup** | **n** | **aOR (95% CI)** | **P value** | **Interaction aOR (95% CI)** | **Interaction P** |
| --- | --- | --- | --- | --- | --- |
| **Overall (primary analysis)** | 246 | 2.43 (1.17–5.04) | 0.02 | — | — |
| **By parity** | | | | | |
| Primiparous | 114 | 6.66 (1.01–43.85) | 0.05 |  | |
| Multiparous | 132 | 1.95 (0.80–4.72) | 0.14 | 0.29 (0.02–5.15) | 0.37 |
| **By maternal education** | | | | | |
| Secondary or below | 113 | 1.41 (0.44–4.48) | 0.57 |  | |
| Diploma/degree or above | 133 | 5.42 (1.62–18.13) | 0.01 | 3.85 (0.33–44.46) | 0.28 |

Note: aOR = adjusted odds ratio. All models used generalised linear mixed models (GLMM) with logit link, random intercept for participant, adjusted for study site, maternal education (or parity in education-stratified models), return to work postpartum, intention to breastfeed, and breastfeeding support. Interaction p-values are from models including a subgroup * intervention interaction term. Stratified models had convergence warnings (large eigenvalue ratios) due to reduced subgroup sample sizes; results should be interpreted with caution. The trial was not powered for subgroup interaction tests, and these analyses are exploratory.

**eTable 4. Proportion of women scoring** $\boldsymbol{\geq}$ **10 on the EPDS at each timepoint**

|  | Control  n (%) | Intervention  n (%) | p |
| --- | --- | --- | --- |
| EPDS $\geq$ 10 at each time point | | | |
| Baseline | 23/123 (18.7) | 27/123 (22.0) | 0.53 |
| 4 weeks postpartum | 24/89 (27.0) | 19/82 (23.2) | 0.57 |
| 8 weeks postpartum | 15/77 (19.5) | 4/70 (5.7) | 0.013 |
| Threshold crossing: Baseline to 8 weeks | | | |
|  | Control (n = 77) | Intervention (n – 70) |  |
| Stayed < 10 (well) | 55 (71.4) | 57 (81.4) |  |
| New onset $\geq$ 10 | 6 (7.8) | 1 (1.4) |  |
| Recovered to < 10 | 7 (9.1) | 9 (12.9) |  |
| Remained $\geq$ 10 | 9 (11.7) | 3 (4.3) |  |

Abbreviation: EPDS, Edinburgh Postnatal Depression Scale. Screening threshold of ≥10 indicates probable postpartum depression. P-values from chi-square tests (uncorrected). Threshold crossing analysis restricted to participants with non-missing EPDS data at both baseline and 8 weeks. Absolute risk difference for screening positive at 8 weeks: 13.8 percentage points; NNT = 7; Cohen d = 0.60.

**Figure S1**

**Figure S1. Normality of continuous outcomes was assessed using Q-Q plots and the Shapiro-Wilk test at each time point.** EPDS scores showed statistically significant departure from normality at all time points in both groups (Shapiro-Wilk p = 0.020 to p < 0.001), with right skew visible on Q-Q plots consistent with a floor effect on depressive symptom scores. BSES-SF scores were approximately normally distributed across all time points and groups (Shapiro-Wilk W = 0.971–0.993, all p > 0.05), supported by Q-Q plots showing data closely following the reference line. BSRQ scores were normally distributed at baseline (p = 0.11–0.68) but showed mild departure from normality at 4 weeks (intervention p = 0.007) and 8 weeks (control p = 0.004, intervention p = 0.033), with slight compression in the tails on Q-Q plots
